# Diameter-driven variation in wood CO_2_ efflux across the stems and crowns of three temperate broadleaf tree species

**DOI:** 10.1093/treephys/tpag060

**Published:** 2026-05-07

**Authors:** Kieran Walker, Alexander Shenkin, Yadvinder Malhi

**Affiliations:** Environmental Change Institute, University of Oxford, South Parks Road, Oxford OX1 3QY, UK; School of Informatics, Computing, and Cyber Systems, Northern Arizona University, Flagstaff, AZ 86011, USA; Environmental Change Institute, University of Oxford, South Parks Road, Oxford OX1 3QY, UK

**Keywords:** carbon metabolism, ecosystem ecology, eco-physiology, scaling, trees, Wytham Woods

## Abstract

A major constraint on modelling woody-tissue CO₂ efflux is the scarcity of datasets that adequately represent its variability and underlying drivers. We intensively sampled CO₂ efflux along the vertical profile of nine temperate broadleaf trees across three species and found substantial longitudinal variation. We partitioned the flux into growth and maintenance-associated components; accordingly we use ‘efflux’ for measurements and ‘respiration’ only for the derived components. Expressing efflux by wood volume, surface area and sapwood volume each offered insight into the underpinning of the flux. However, efflux was most strongly associated with surface area, particularly in medium and large branches, and across both growth and maintenance respiration. In small branches, where sapwood dominates, this surface area scaling weakens, suggesting maintenance respiration shifts from surface area- to volume-based scaling, while growth respiration remains associated with surface area. Sapwood depth, tree ring width and the relative contributions of growth and maintenance respiration explained much of the variation in efflux, though additional biological and physical factors, such as CO₂ diffusion from sap and variation in sapwood parenchyma, likely also contribute. These findings represent an important step towards reducing uncertainty in the spatial scaling of woody-tissue CO₂ efflux by linking within tree variation in respiration to underlying anatomical and growth-related controls.

## Introduction

Ecosystem respiration worldwide emits ~ 60 Gt of CO_2_ to the atmosphere annually. The size of this flux relative to photosynthetic draw-down equates to the strength of the biosphere as a carbon sink (Grace 2004). As much as 40–70% of this flux stems from plant tissue (i.e., autotrophic respiration) which is itself governed by both the physiological requirement of plant tissue for energy and by environmental factors such as temperature, drought and soil type (DeLucia et al. 2007, Campioli et al. 2016). As a result, respiration varies widely across time and space. Understanding this variation is critical to the development of land surface models but is complicated by the independent responses and physiology of each respiration component, i.e., leaf, woody-tissue and soil (Cramer et al. 2001, Noh et al. 2024). As such, a mechanistic understanding is needed of each sub process of autotrophic respiration in order to model ecosystem–atmosphere interactions under future climatic conditions.

The woody-tissue component accounts for between 5% and 40% of autotrophic respiration across forested biomes (Rodríguez-Calcerrada et al. 2014, Campioli et al. 2016, Yang et al. 2016), but there is large uncertainty around those estimates largely attributed to our inability to quantify important scalars such as woody surface area and volume. Woody respiration has often been implicated as a source of variation in net primary productivity (NPP) between stands and seasons (Ryan et al. 1994, DeLucia et al. 2007, Robertson et al. 2010, Campioli et al. 2016). Most commonly measured by the molecular evolution of CO_2_ over time using infrared gas analysers (IRGAs) applied directly to a tree’s bark surface, a measure of the efflux rate at specific points can then be multiplied by some metric of tree structure to yield the woody-tissue contribution to ecosystem respiration (Yoda et al. 1965). Despite its importance in forest carbon dynamics, a granular understanding of woody-tissue respiration has lagged that of other components such as leaves and soil due to the functionally diverse physiology of woody tissue and the difficulty of characterizing its structure. This has resulted in the persistence of large estimate uncertainties (30–50%) when scaling up this flux (Metcalfe et al. 2010, Meir et al. 2017). This study aims to address the biological underpinnings of the woody-flux and key uncertainties in scaling woody-respiration in a broadleaf English forest and thereby identify potential widespread bias in ecosystem carbon flux.

Early studies of woody-tissue respiration demonstrated substantial variation in the rate of efflux between individuals, and more recent work has highlighted vertical variation within individuals (Ryan et al. 1994, Cavaleri et al. 2006, Katayama et al. 2019). However, interpreting these data is complicated by the fact that CO_2_ efflux from bark is not solely the result of the adjacent respiring tissues; it also includes a variable fraction originating in soils and roots, and that even the fraction originating from woody tissue is a function of distinct biological and environmental processes (Chambers et al. 2004, Bowman et al. 2005, Salomón et al. 2020). Attempts to understand these variations have often focussed on the metabolic requirement for energy at the individual level, i.e., the mass of cells to be energetically supported, namely the parenchymal content, and the rate and energetic cost of constructing biomass (Meir and Grace 2002, West 2020). Such studies have successfully looked to sapwood content (or stem diameter by proxy) and radial stem growth to explain between-tree variation in terms of maintenance and growth respiration, respectively (Sprugel and Benecke 1991, Lavigne et al. 1996). These parameters also vary within individuals, and accordingly variation in CO_2_ efflux has been observed within individuals although no consensus has been reached as to the extent or patterning of this variation (Cavaleri et al. 2006, Araki et al. 2010, Katayama et al. 2014, 2019, Westerband et al. 2022). Such within-individual variation is a problem for the validity of most previous estimates of ecosystem woody respiration, which have typically applied a singular rate as measured at the bottom of a tree across all branch tissue. This practice could result in erroneous estimates if rates of efflux from the upper branches are, on average, different from the basal measurement.

A key unsettled issue presented by moving from point-based measurements of CO_2_ efflux to whole-tree and stand estimates is the selection of an appropriate scaling parameter. Total mass, nitrogen content and crown span have all been applied, but the most utilized are surface area, total volume and sapwood volume (Yoda et al. 1965, Ryan et al. 1994, Yokota et al. 1994, Levy and Jarvis 1998, Stockfors and Linder 1998, Mori et al. 2010, Collalti et al. 2020, Westerband et al. 2022, Mills et al. 2025). Scaling by sapwood volume is ostensibly the most mechanistic approach for maintenance respiration since sapwood contains the most living woody tissue, although questions persist about the uniformity of metabolism and distribution of parenchyma cells (Spicer and Holbrook 2007, Ziemińska et al. 2013, Rodríguez-Calcerrada et al. 2015). Furthermore, sapwood volume’s utility as a scalar is limited not only by the fact that sapwood metabolism is variable within an individual depending on the parenchymal content therein but also by its reliance on difficult-to-collect data (Shinozaki et al. 1964). Whole-tree tissue volume and surface area, also difficult to measure until recently (Lau et al. 2018, Malhi et al. 2018), have both been used as scalars, although no consensus has been reached as to the efficacy of one over the other. Studies report varied success between species and branch size classes (Meir and Grace 2002, Katayama et al. 2016). This likely represents physiological differences in tissue composition. Theory dictates that CO_2_ efflux will scale with surface area when the flux originates from thin tissues localized close to the surface of the tree (e.g., vascular cambium; sapwood on branches dominated by heartwood) and will scale with volume when originating from tissues spread evenly throughout the depth of the stem or branch (e.g., sapwood dominated branches, absent of heartwood). We can therefore expect both maintenance and growth respiration to scale with surface area in trunks and larger branches provided that both sapwood and cambium quantity and activity remain relatively constant. By the same logic, in the very-small diameter branches where heartwood is yet to develop, we expect maintenance respiration to favour volume-based scaling due to the presence of live tissue throughout the branch cross-section (for a schematic, see Figure S1 available as Supplementary Data at *Tree Physiology* Online in supplement). However, it should be noted that variable cambial activity and parenchymal content within sapwood, as has previously been reported for some species (Morris et al. 2016), may potentially obscure or produce non-linear relationships of respiration with branch diameter.

The growth component of respiration is a significant, albeit variable fraction of the woody-tissue respiration and is a key source of uncertainty in models of NPP and gross primary productivity (Dietze et al. 2014, Jardine et al. 2022). Differences in growth rates have long been used to explain variation in rates and scaling relationships of CO_2_ efflux over time and between individual trees (Rodríguez-Calcerrada et al. 2014, Jardine et al. 2022, Mills et al. 2024) but few studies have considered how this might vary between different points within trees despite evidence that rates of growth are greater in branches than in stems and boles (Tarvainen et al. 2014, Han et al. 2017). Previous work addressing within-tree variation in growth has utilized dendrometer bands to continuously monitor incremental increases in diameter over time; however, this is logistically difficult and requires repeated journeys into the canopy to install and collect data and has resulted in growth rates only being considered at a limited number of locations within a tree. This is problematic because the application of these scaling relationships and growth rates, as measured near the ground, to the branches of the canopy will result in inaccurate models of forest carbon accumulation and exchange, especially given that the majority of a trees biomass may be stored in the branches (Demol et al. 2021).

In this study, we present a more thorough investigation of within-tree variation in rates of CO_2_ efflux than any previous work we are aware of, making point measurements at between six and eight locations covering the full range of diameters on each of nine individual trees across three species in a temperate broadleaf forest. Unlike earlier studies that either profiled efflux along the stem (e.g., Tarvainen et al. 2014) or partitioned growth-vs-maintenance at diameter at breast height (DBH) (Rodríguez-Calcerrada et al. 2019), our dataset combines six to eight diameters per tree from base to crown, and matched increment cores for tree-ring width and sapwood depth at every efflux point allowing us to investigate how respiration and its growth and maintenance components vary across branch diameters. We also present data from a larger number of trees of two species at breast height to broaden our understanding of sources of variation between individuals.

Our key objectives and questions are as follows. (i) How do rates of CO_2_ efflux from tree stems and branches vary with branch diameter within individual trees and across species? (ii) Which structural scaling parameter best captures this variation and what does that tell us about ecophysiological controls on respiration? (iii) How do growth and maintenance components of respiration vary between and within individuals and two species?

## Materials and methods

### Site description

Data were collected at Wytham Woods (1°20 W, 51°47 N), a semi-natural research woodland to the northwest of Oxford owned by Oxford University (Savill et al. 2011). It is considered a category of W8 *Fraxinus excelsior*–*Acer campestre*–*Mercurialis perennis* woodland in the UK National Vegetation Classification (Rodwell 1998). Mean annual rainfall is 726 mm and is distributed evenly throughout the year. The mean annual temperature is 10 °C and mean annual solar radiation is 118 W m^−2^ (Centre for ecology and hydrology (CEH) and Morecroft et al. 1998). The forest immediately surrounding the specific site of targeted sampling is defined as disturbed ancient woodland (woodland has not been cleared since at least the 1600s; Peterken and Game 1981). Stem density in Wytham is 1128.2 ha^−1^, with a basal area of 31.6 m^2^ ha^−1^. The peak leaf area index was calculated as 7.8 m^2^ m^−2^ in 2008 and the upper and lower mean canopy height is 17 and 6.9 m, respectively. The soil texture has been characterized as: clay 60%, silty clay 22%, clay loam 15%, silty clay loam <5% (Butt et al. 2009, Fenn et al. 2010).

Sampling was conducted between June and August 2017, with the majority of measurements made in July. A second campaign constituting 1 day of sampling was conducted in July 2020 to increase the number of observations specifically at <5 cm diameter. Sampling was targeted towards the three most dominant species at the site, *Acer pseudoplatanus* L (sycamore), *Quercus robur* L (English oak, hereafter oak) and *Fraxinous excelsior* L (European ash, hereafter ash). These species together make up 90.1% of basal stem area in the wood due to their abundance and large size (Butt et al. 2009). Consequently, they are the predominant sources of woody autotrophic respiration and represent important stores of carbon in this ecosystem.

### Sampling design

The design included a targeted sample of three individuals each of ash, oak and sycamore trees which were sampled intensively along a vertical transect at six to eight locations depending on tree size. Starting at 1.3 m above the ground, measurements were made mid-way between all major bifurcations following the most upright limb until the upper canopy was reached. For the purpose of this intensively sampled data set, stem diameter is treated as the largest diameter within a tree, i.e., the first measurement of each tree, after which all measurements refer to branches. A further 25 individuals of ash and oak were sampled at breast height (1.3 m) in order to investigate variation of growth rates and respiration between individuals as well as within individuals.

Intensively sampled trees were selected according to several criteria: that they were healthy individuals showing no obvious signs of decay; and that they were deemed safe for climbing. Trees were sampled so that a range of sizes were included (small, medium, large), the diameters of these intensively sampled trees at breast height ranged from 49.3 to 142.6 cm and their heights ranged from 15 to 25 m.

The intensively sampled trees were sampled between 19 June and 2 July, with each tree generally taking a whole day to sample due to the logistics of accessing the crown. Measurements were made between 10 a.m. and 5 p.m. to avoid large changes in ambient temperature. A single point measurement consisted of quantifying CO_2_ efflux from the surface of stems and branches using an IRGA subsequently collecting a wood-core from same tissue to which the IRGA was applied. Alongside these, air and stem temperature below the cambium was recorded using a copper-constantan thermocouple and the diameter of the limb was recorded.

For diameters and corresponding heights and temperatures, see Table S1 available as Supplementary Data at *Tree Physiology* Online.

### Quantifying CO_2_ efflux

An IRGA (EGM-4 and SRC-1 soil chamber, PP Systems, Hitchin, UK) was employed to quantify CO_2_ efflux directly from the bark (Marthews et al. 2014). This IRGA fits readily over a 105 mm wide, 50 mm deep PVC collar. For the larger branches and stems, this collar was easily fit to the wood with a commercially available putty (Plumbers Mait, Bostik Ltd, UK) to achieve an air-tight seal. The putty was tested for outgassing. For smaller branches and stems, a collar with a reduced diameter was required to achieve a closed seal between the IRGA and tree. For this a modified plastic funnel, fit with a small fan to ensure adequate air mixing and a pipe joiner that matched to a 4.6 mm diameter PVC pipe was sealed to the tree in the same way as the larger collar. The smallest branches (<50 mm diameter) of the upper canopy were excised for measurement after the ends had been sealed with cling film and electrical tape to avoid enhanced CO_2_ diffusive loss through the cut ends and then placed in a chamber built from a 400 mm long, 105 mm diameter pipe fit with a fan to ensure adequate air mixing.

The effect of temperature variation on CO_2_ efflux was compensated for by measuring sapwood temperature with a K-type thermocouple inserted into the sapwood via a slit created by a pocketknife. The well-described effects of temperature on CO_2_ efflux were accounted for by normalizing rates to 23 °C, the average daytime sapwood temperature during the period of study using a *Q*_10_ correction factor of 2, which is the most common Q_10_ value prescribed to woody tissue in the literature (Ryan 1991, Meir and Grace 2002,  Wang et al. 2006. Furthermore, given that the effect of temperature on metabolic rates can exhibit a lag of some hours (Ryan et al. 1995, Salomón et al. 2024), sampling was conducted between 10 a.m. and 5 p.m. to avoid large variations in temperature.

### Wood core collection and analysis

After respiration measurements, increment cores were collected from the stem or branch section using a 5 mm increment borer (Haglöf, Sweden). These samples were drawn from the exact same location as the corresponding measurement of respiration. The borer was inserted into the tree roughly 90° to the stem or branch with a slight upward angle to prevent water ingression into the bore hole, thus minimizing the risk of fungal tree infection. Where trees were leaning on an angle, samples were taken at 90° to the direction of the lean to avoid sampling errors from reaction wood.

Sapwood and heartwood have diverse characteristics across species and cannot be demarcated by one single method. This was a persistent problem throughout this study and in sycamore we were unable to find an effective method. In the case of most oak samples, the sapwood and heartwood were delineated by visible colour change, and where the boundary was not clear, methyl orange indicator solution was painted onto the wood cores. This turned the heartwood a deep red colour and left the sapwood orange owing to differences in acidity; oak heartwood has a lower pH than its sapwood due to higher a concentration of phenol groups (Miranda et al. 2017). In ash, heartwood was distinguished from sapwood based upon the abundance of tyloses in occluded xylem vessels; a process that occurs as sapwood is converted to heartwood in order to prevent the transport of pathogens via water flow throughout the heartwood (Chattaway 1952). Vanillin and iodine, used to detect starch in live cells, were ineffective in all three species.

Dendroecology and the study of ring widths can be used to gauge annual variation in growth rates in so called ring porous species which have considerably larger vessels in early wood compared with late wood of the same season (Amoroso et al. 2017). Wood cores were photographed next to a ruler and measured digitally using ImageJ software (Schneider et al. 2012) for cores from oak and ash but in the case of sycamore, which is a ring diffuse species with no obvious ring development, we attempted the measurement but found that it was not possible.

### Dendrometer data and analysis

Dendrometer data are described in Butt et al. (2009). In order to scale growth rates across seasons, dendrometer data were used to provide a measure of the seasonality of woody-tissue growth. Seasonality was modelled on a species-specific basis and combined with the absolute growth rates of individual trees, as measured by ring widths and diameter increases between censuses. This provided a measure of both the amount and seasonality of woody-tissue accumulation in our sample.

### Data analysis

#### The relationship between branch and stem diameter and CO_2_ efflux

Data analysis and visualization was conducted in R (R Core Team 2023).

Temperature compensated CO_2_ efflux was expressed as a function of three scalars: (i) woody surface area, (ii) wood volume subtending the measurement (approximated as the volume of a cone with a base equal to measurement area and height equal to branch or stem radius) and (iii) sapwood volume (approximated as the volume of a truncated cone). The goal was to explain as much variation in CO_2_ efflux as possible using parameters extractable from TLS-derived quantitative structural models (Raumonen et al. 2013) as this is the best option for scaling in the near future (Meir et al. 2018).

Using linear-mixed models (R package lme4; Bates et al. 2015) and generalized additive models (GAMs; R package *mgcv*; Wood 2017), CO_2_ efflux per volume and bark surface area was modelled as a function of branch diameter to investigate the explanatory capacity of each. Branch height was also investigated but was non-predictive, even as a component of mixed regression models. GAM smoothing functions were selected by restricted maximum likelihood using Gaussian family error structures. The number of basis functions included was determined by selecting for models based upon the variance explained and Akaike Information Criterion (AIC) values. Model residuals were tested against a null distribution and visualized to ensure that they were randomly distributed as per the assumptions of the model.

The existence of a scaling relationship of respiration with surface area and volume can be tested by expressing the temperature-corrected flux both by the area (Rt^area^) and the volume (Rt^vol^) subtending the measurement and observing its relationship with branch diameter (Levy and Jarvis 1998). If a perfect scaling relationship with surface area holds, then Rt^area^ (i.e.*,* the flux divided by the sampled area), will be constant across branch diameters because whilst the volume of tissue subtended is changing, the surface area is not. Furthermore, if area scaling holds then Rt^vol^ (i.e.*,* the flux divided by volume subtended) should exhibit a strong negative response to increasing branch diameter with rates of efflux being highest in the smallest diameter branches where surface area-to-volume subtended ratios are greatest, and lowest in the largest diameter branches where surface area-to-volume subtended ratios are lowest. Conversely, if the flux scales perfectly with branch volume, then Rt^area^ should increase linearly with branch diameter as the volume of respiring tissue increases but the area measured remains constant. Additionally, if volume-based scaling holds, then Rt^vol^ should yield no relationship with diameter because as volume increases any effect is negated by dividing the flux by the increasing subtended volume.

### Separating growth and maintenance respiration

Two methods were applied to calculate the relative contributions of growth and maintenance respiration as per the methods of Meir and Grace (2002). Both methods assumed our measured fluxes to be a combination of both growth and maintenance respiration and aimed to determine the relative contributions of each component by calculating one and subtracting it from the total to leave the other component. Woody-tissue growth is known to primarily occur by the division of cambial cells, the cambium being a laminar tissue that is generally only two cells thick. Therefore, in calculating the growth fraction, fluxes were expressed by area even if maintenance respiration was subsequently expressed by volume.

The first method (the construction cost method) involved calculating the metabolic cost in grams of CO_2_ of constructing new woody tissue directly under the IRGA measurement for each individual tree and considering this to be the annual growth flux. This was then subtracted from the total flux to yield individual maintenance respiration. The metabolic cost of wood in grams of CO_2_ was empirically derived by Penning De Vries (1975) as 0.43 g CO_2_ per gram new wood mass. Using the incremental increase in diameter over 6 years the volume of wood grown under the IRGA measurement was calculated for each individual and scaled by a species-specific seasonal growth curve to provide the incremental increase in the month CO_2_ efflux was measured. This volumetric increase, multiplied by the species-specific gravity of wood, provides the mass of new wood at each measurement. Multiplying values for new wood mass by 0.43 therefore yields the amount of CO_2_ expended growing woody tissue and provides the instantaneous growth flux in grams of CO_2_.

A second method (the regression-based method) was applied to validate the above approach. In regressing the total measured flux against volumetric increase of woody tissue beneath the IRGA collar (as calculated for the construction cost method) on a species-specific basis, the *y* intercept of the model can be considered the sample average rate of efflux at zero growth, i.e., the sample average maintenance respiration. Average maintenance respiration was represented as a fraction of total respiration and the difference between the two was considered the fraction of growth respiration.

### Inter-annual and intra-annual variation in growth rates

The growth rate of trees in Wytham Wood is known to vary seasonally and between species (Fenn et al. 2010). Having been measured in two subsequent censuses the absolute amount of growth over 6 years was known for each tree on which CO_2_ efflux was measured. In order to separate the growth and maintenance fractions of respiration as per the above methods, the instantaneous rate of growth at the time CO_2_ efflux was measured was needed. This was derived by developing species specific growth curves for each year between 2011 and 2017 (Figure S2 available as Supplementary Data at *Tree Physiology* Online in supplement) and scaling tree specific total tree growth by these curves to calculate the individual specific growth rate in June 2017—when CO_2_ efflux was measured.

Species-specific growth curves were developed using monthly diameter measurements made by dendrometer data for 350 and 133 individuals of ash and oak, respectively, over the duration of the growing season. To these data, sigmoidal curves were fit that represented the cumulative growth of each individual tree on a yearly basis from 2011 to 2017. These sigmoidal curves were then converted to monthly changes in diameter, essentially providing the seasonal growth curve of each individual tree. These were then pooled by species and fit with GAM splines to provide the species average seasonal growth curve. These curves were then used to scale the specific tree growth over 6 years to the rate of growth in June 2017 by dividing total increment increase by the area under all growth curves and multiplying the product by the growth rate in June 2017.

Because dendrometer data were not available for some of the specific trees on which we measured CO_2_ efflux, a separate sample of the same species from within Wytham Wood was used. In order to assess whether the two samples followed similar growth patterns, the average annual growth rates as measured by dendrometers were regressed against average ring widths from the respiration trees for the corresponding year. This allowed us to assess the extent to which the two growth-estimation approaches produced comparable results.

### Diameter-dependent variation of growth rate

Using linear and GAMs (R package *mgcv*; Wood 2017) average ring width was modelled as a function of branch diameter in ash and oak. Because cores were extracted mid-growing season and the first ring often separated from the underside of the bark during extraction, likely resulting in un-quantified damage to an already exceedingly small and difficult to measure growth ring, the outermost ring was excluded from the dataset. For this reason, when investigating the diameter dependence of variation in growth rates, the mean of six ring widths was used. Furthermore, in scaling the growth and maintenance component of respiration across branch diameters within trees, the difference of mean ring width relative to mean DBH ring width was used.

## Results

### Diameter-dependent variation of respiration

**Figure 1:**
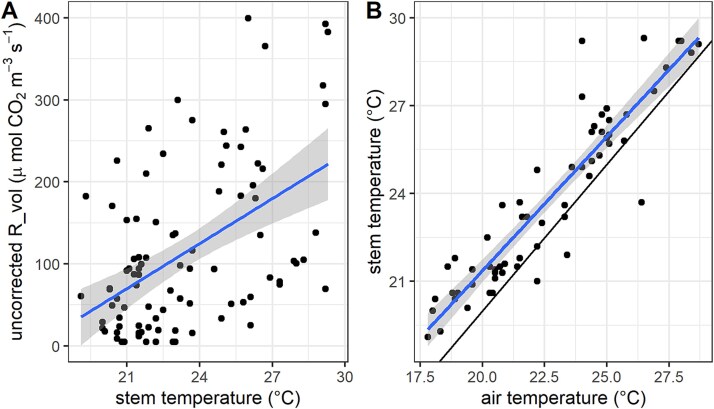
(A) The effect of stem temperature as measured below the cambium on woody-tissue CO_2_ efflux from bark (*r*^2^ = 0.24, *P* < 0.000). Data are pooled across nine trees (three per species) and include measurements from both stems and branches. (B) The relationship of sapwood and air temperature over the period of study (*r*^2^ = 0.85, *P* < 0.001). Black line = 1:1.

**Figure 2:**
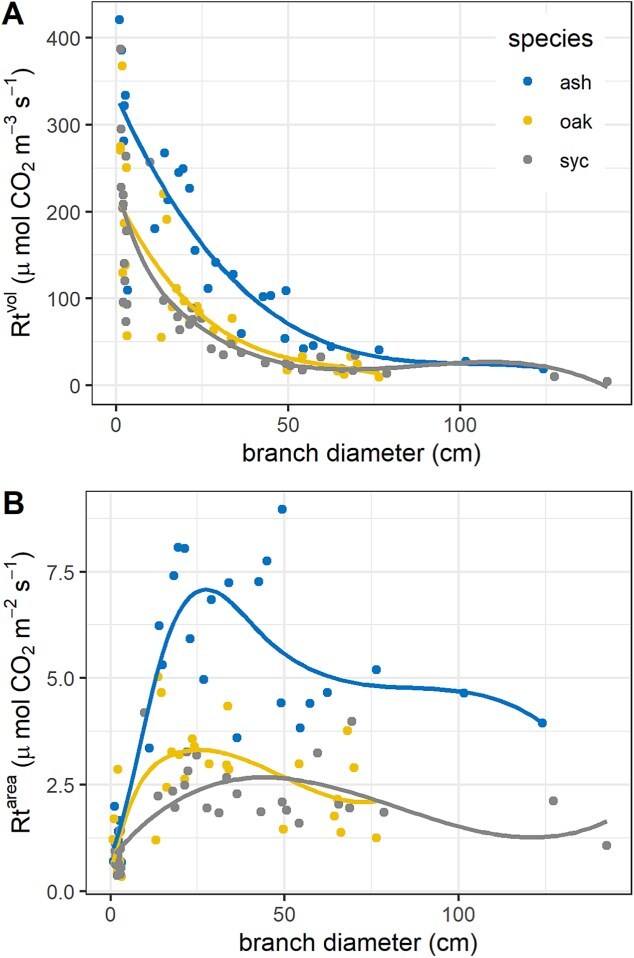
Relationships between branch diameter and woody-tissue respiration expressed per unit volume (Rt^vol^; A) and per unit surface area (Rt^area^; B) in ash, oak and sycamore (*n* = 9 trees, 3 per species). Data include measurements from stems and branches spanning the full vertical profile from the stem base (1.3 m) to the canopy. Lines show species-specific GAM smooths fitted to the data.

**Figure 3:**
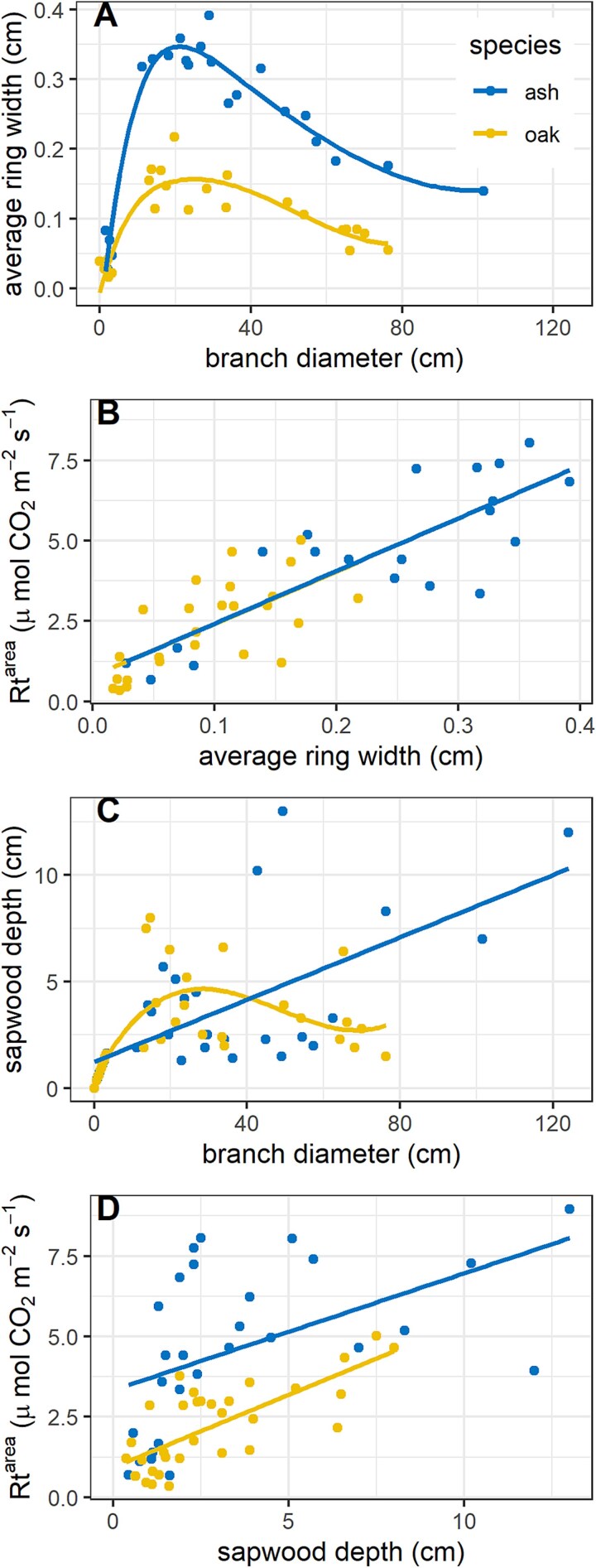
(A) Mean ring width in relation to branch diameter in oak and ash, with GAM smooths fitted to the data. (B) The relationship between Rt^area^ and mean ring width in ash and oak. (C) The relationship between sapwood depth and branch diameter in ash and oak. (D) The relationship between Rt^area^ and sapwood depth in ash and oak (*n* = 6 trees, 3 per species).

Mean measured CO_2_ efflux from woody tissue across all nine intensively sampled individuals was 3.71 ± 2.20 μmol m^−2^ s^−1^ and ranged from 0.75 to 8.83 μmol m^−2^ s^−1^. Over the period of study, mean sapwood temperature was 22.06 ± 3.10 °C, whilst mean air temperature was 21.10 ± 2.06 °C. Sapwood and air temperature were highly correlated, though sapwood temperatures were generally 1–2 °C warmer (Figure 1B). Using ordinary least squares regression (OLS) on pooled data, a significant effect of stem temperature on raw CO_2_ efflux was found (*y* = −609.67 + 32.19 *x*, se = 136.96, *r*^2^ = 0.23, *P* < 0.001). However, after normalizing these data to a reference diameter and growth rate (the median of the sample), this temperature effect was no longer apparent. Rates of efflux were therefore normalized to the average stem temperature (Rt) during the period of study, using literature values for *Q*_10_ (see Materials and methods). The mean diameter of sampled stems and branches was 38.56 ± 30 cm and ranged from 3.69 to 142.61 cm (Figure 2 and 3).

A combined GAM model (Table S2d available as Supplementary Data at *Tree Physiology* Online) including all three species indicated that, after controlling for diameter effects, ash trees had significantly higher rates of CO_2_ efflux than did sycamore and oak both when expressed by volume (^vol^) and by area (^area^) subtending the measurement (Tables S2d and S3d available as Supplementary Data at *Tree Physiology* Online). Species-specific GAMs of CO_2_ efflux as a function of branch diameter were significant for all species (Figure 2); however, models of Rt^vol^ were more predictive than Rt^area^, with Rt^vol^ explaining 71.1%, 59.1% and 65.1% as opposed to 50.4%, 52.7% and 46.5% by Rt^area^ for ash, oak and sycamore, respectively (Tables S2 and S3 available as Supplementary Data at *Tree Physiology* Online). Furthermore, the direction of the effect on CO_2_ efflux differed between the two scalars. Expressing CO_2_ efflux by the volume of all tissue subtending the measurement (Rt^vol^, Figure 2A), a strong negative effect of branch diameter was found with smaller diameter branches having higher rates of efflux relative to their volume than their larger counterparts. In contrast, Rt^area^ (Figure 2B) exhibited a positive effect of diameter on efflux over samples <20 cm diameter, followed by a plateau.

A combined GAM including both ash and oak found that after controlling for diameter effects, the growth rate of ash, as measured by ring width, was significantly higher than that of oak (Figure 3A and Table S4a available as Supplementary Data at *Tree Physiology* Online). The GAM models of both ash and oak indicated that ring width was significantly related to branch diameter (deviance explained by diameter: ash = 88%, oak = 79%). The smallest ring widths were observed at the smallest diameters and increased quickly over diameters of 3–20 cm before decreasing steadily in the medium and larger diameter branches.

An ordinary least squares regression demonstrated that mean ring width was significantly and linearly related to Rt^area^ (Figure 3B). This relationship held for both ash and oak with no difference in the slope or intercept of the effect detected (*y* = 0.79 + 16.35 *x*, *r*^2^ = 0.65, *P* > 0.001; *y* = 0.79 + 16.21 *x*, *r*^2^ = 0.43, *P* < 0.001, ash and oak, respectively).

Sapwood depth was linearly related to diameter in ash (*y* = 1.23 + 0.07 *x*, *r*^2^ = 0.41, *P* < 0.001) but in oak required GAM modelling to characterize the relationship (deviance explained by smooth of diameter = 43%, Table S5 available as Supplementary Data at *Tree Physiology* Online), which increased with diameter up to ~30 cm before plateauing and decreasing slightly in the larger diameter branches (Figure 3C). Ordinary least squares regression revealed that sapwood depth underlying the measurement had a significant positive effect on Rt^area^ in both ash (*y* = 3.33 + 0.36 *x*, *r*^2^ = 0.22, *P* = 0.006) and oak (*y* = 0.93 + 0.45 *x*, *r*^2^ = 0.53, *P* < 0.001), with deeper sapwood resulting in larger fluxes (Figure 3D).

### Seasonal variation in growth rates: Dendrometer and ring data

Modelling monthly change in incremental increase of diameters at breast height we see that ash trees on average have a more intense growing season, with greater diameter increases observed at the peak of growing compared with oak. However, we also observe that the length of the growing season is longer in oak in comparison with ash and that in both species we see some inter-annual variation with regards to the month in which growth rates peak (Figure S3 available as Supplementary Data at *Tree Physiology* Online). We also find that the tree growth, as measured by ring width and dendrometer bands, follows the same general inter-annual variation in total growth. Furthermore, we find that the average growth as measured by dendrometer bands can be used to predict the average growth of the sample as measured by ring width (Figure S4 available as Supplementary Data at *Tree Physiology* Online, *y* = 2.45 + 0.25 *x*, *r*^2^ = 0.88, *P* < 0.01), with the intercept of oak being significantly lower than the model average (−1.32, *P* = 0.01), albeit with no significant difference in the slope.

The construction-cost and regression method yielded broadly similar results for the average relative contribution of the growth and maintenance components of respiration (Table 1). Both agree that on an annual basis the maintenance component dominates the flux in both species. In June, however, when growth rates are high, growth accounts for a much larger proportion of the flux, ~ 30–40% in oak and ~48–66% in ash, reflecting the fact that 27% and 36% of annual growth is achieved in this month for oak and ash, respectively.

**Table 1:** Growth and maintenance components of respiration (using data at DBH only). Species average growth and maintenance respiration as calculated using both the regression and construction methods presented for June and per annum (p.a.).

	Percentage share of total respiration					
	Growth (%)	Growth 95% CI	Maint. (%)	Maint. 95% CI	se (%)	Period
Method: regression						
Oak	9.25	−39.23, 47.65	90.75	52.35, 139.23	24.83	p.a.
Oak	30.50	−0.1798, 0.6890	69.50	0.3110, 1.1798	24.83	June
Ash	15.18	−18.61, 56.58	84.82	43.42, 118.61	16.30	p.a.
Ash	66.30	0.3251, 1.0770	33.70	−0.0770, 0.6749	16.30	June
Method: construction						
Oak	11.75	6.92, 18.67	88.25	80.27, 92.69	9.93	p.a.
Oak	39.78	23.40, 63.19	60.22	36.81, 76.60	33.62	June
Ash	11.52	9.24, 12.74	88.48	86.58, 90.27	4.63	p.a.
Ash	48.47	40.33, 55.62	51.53	44.38, 59.67	14.70	June

### Diameter-dependence of the growth and maintenance components of respiration

**Figure 4:**
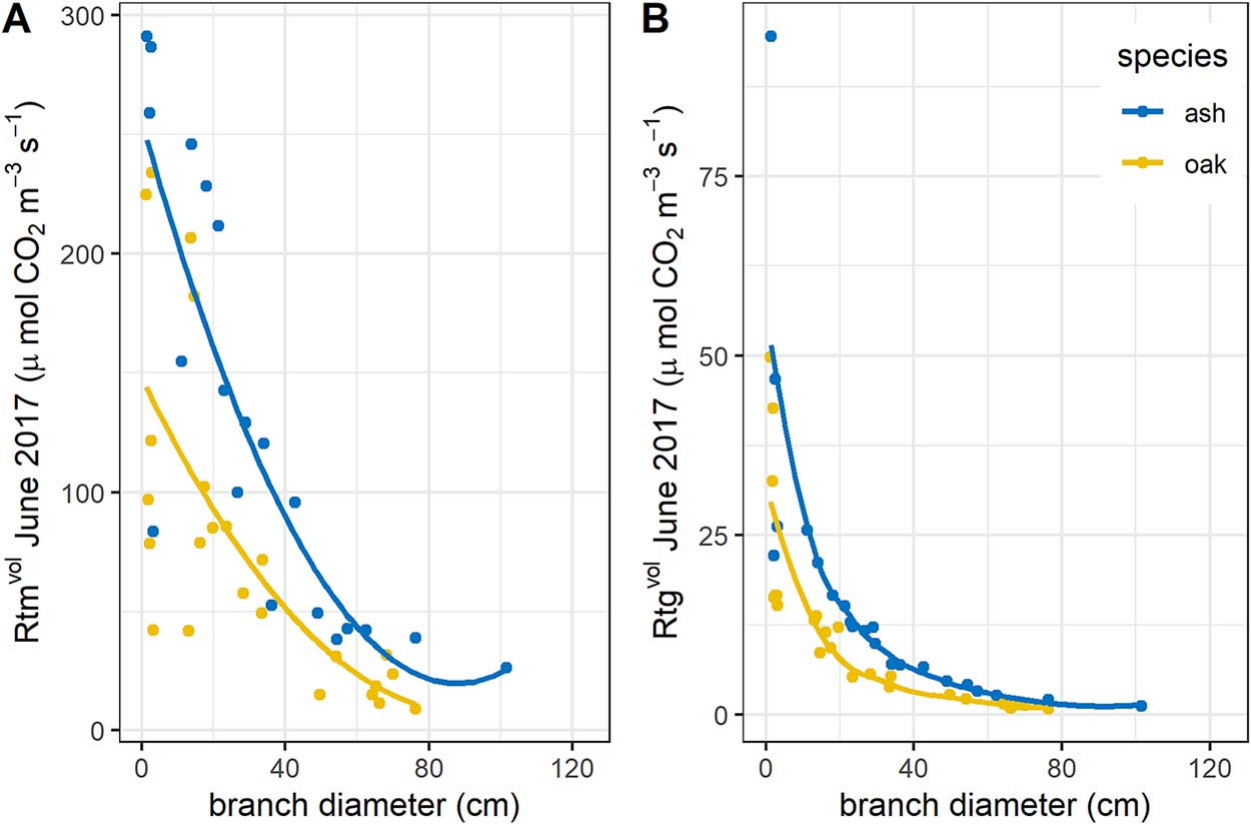
Diameter-related variation in growth (Rtg^vol^; A) and maintenance (Rtm^vol^; B) respiration expressed per unit volume in three trees each of oak and ash. The GAM smooths indicate that diameter explains a substantial proportion of variation in both components but is more predictive in ash (see Table S6 available as Supplementary Data at *Tree Physiology* Online).

**Figure 5:**
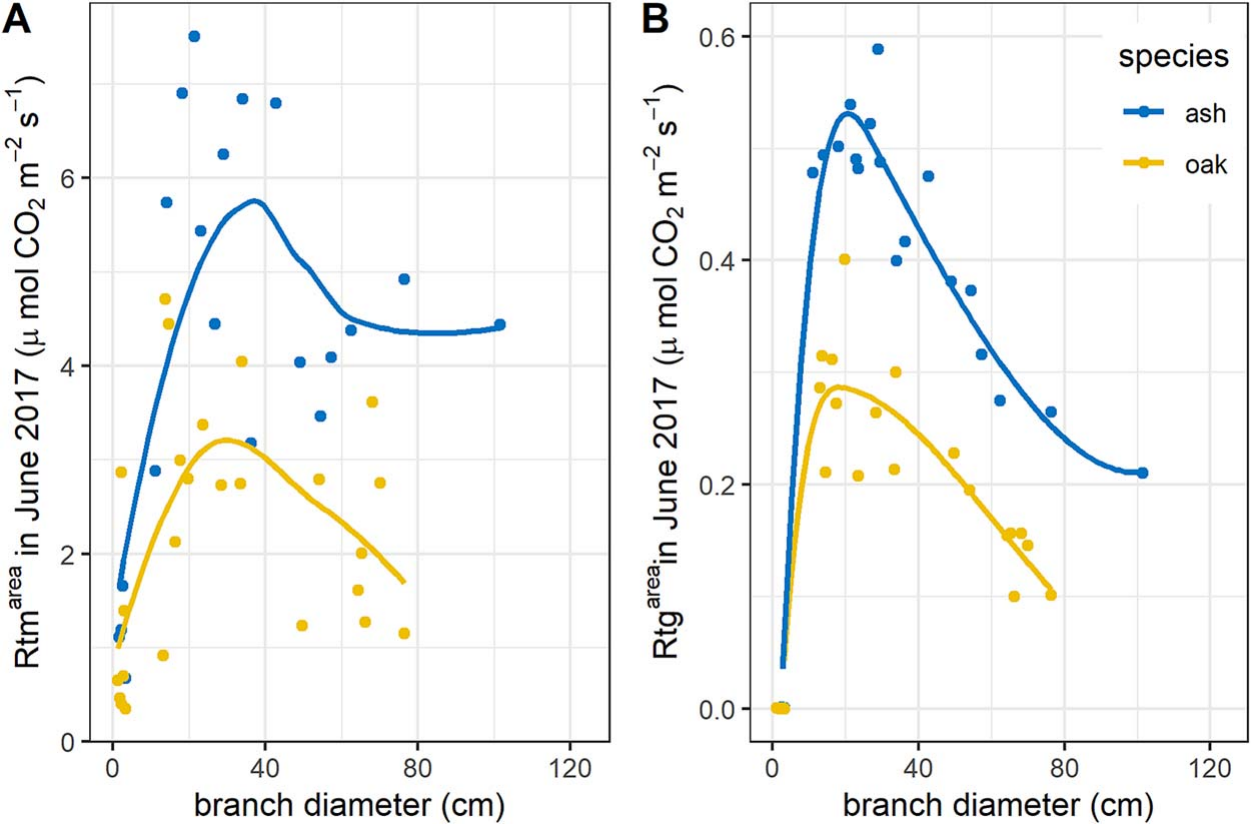
(A) Diameter-dependent variation of maintenance respiration expressed per unit area (Rtm^area^). (B) Diameter-dependent variation of growth respiration per unit area (Rtg^area^). Data are for 3 trees each of ash and oak, lines show GAM smooths fitted to the data (see Table S7 available as Supplementary Data at *Tree Physiology* Online).

Rates of Rtm and Rtg (temperature compensated maintenance and growth respiration, respectively) generally followed the same scalar-specific trends with branch diameter as observed in combined CO_2_ efflux. Rtm^vol^ and Rtg^vol^ were lowest in larger diameter branches and highest in smaller diameter branches (Figure 4; Rtm^vol^ deviance explained by diameter; oak = 49%, ash = 71%. Rtg^vol^, deviance explained by diameter; oak = 69%, ash = 61%). Similarly, Rtm^area^ and Rtg^area^ were lowest in the smallest diameter branches, increasing with diameter before plateauing at ~20 cm diameter and declining slightly in the largest diameter branches (Figure 5; Rtm^area^ deviance explained by diameter; oak = 33%, ash = 30%. Rtg^area^; oak = 68%, ash = 45%). Furthermore, a combined species GAMs indicated that both the Rtm and Rtg were higher in ash than in oak regardless of whether volume or area scaling was used (Tables S6 and S7 available as Supplementary Data at *Tree Physiology* Online).

**Figure 6:**
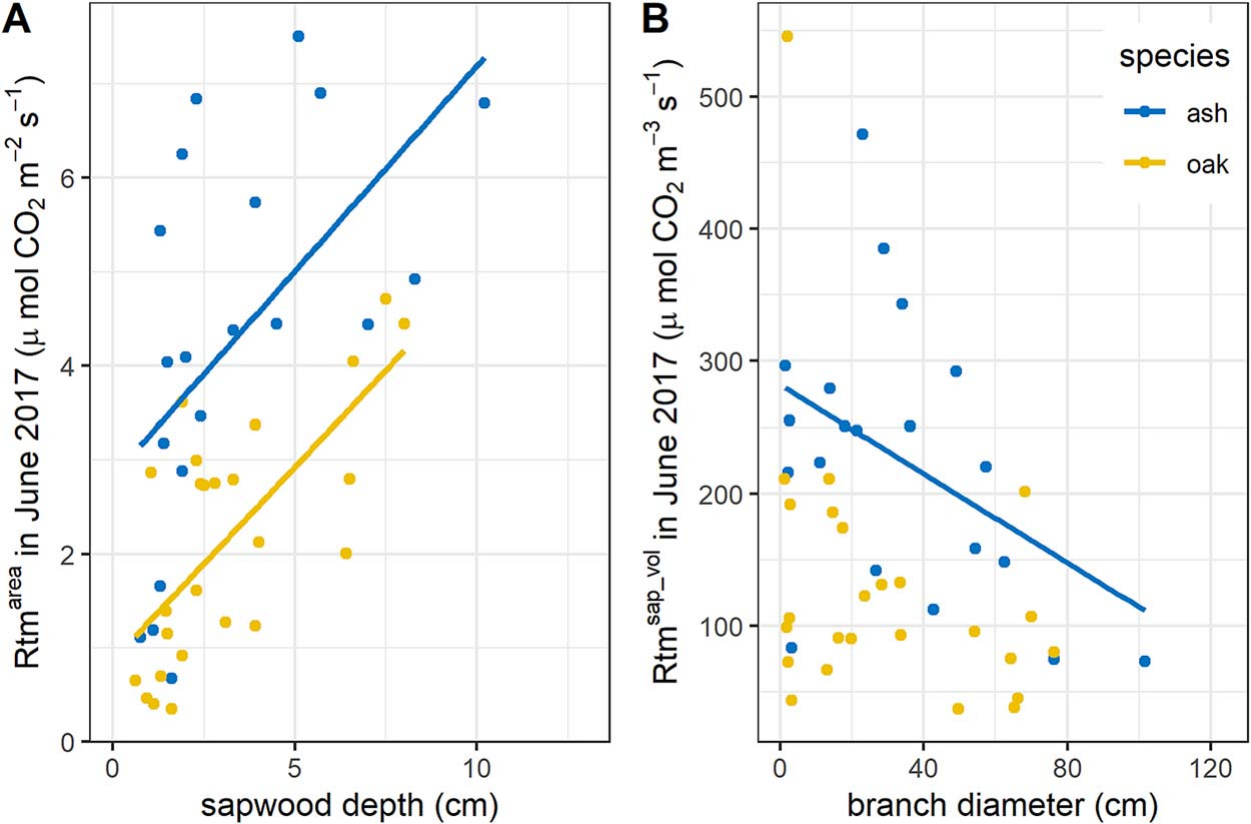
(A) Maintenance respiration expressed per unit surface area (Rtm^area^) scaled to June, in relation to sapwood depth in ash and oak. (B) Maintenance respiration expressed per unit sapwood volume (Rtm^sap-vol^) in relation to branch diameter in ash and oak.

Rtm^area^ was significantly and linearly related to sapwood depth in both ash (Figure 6A; *y* = 2.82 + 0.437 *x*, se = 0.15, *r*^2^ = 0.31, *P* = 0.010^*^) and oak (*y* = 0.868 + 0.411 *x*, se = 0.08, *r*^2^ = 0.47, *P* = < 0.000). Maintenance respiration expressed by the volume of sapwood subtending the measurement (Rtm^sap-vol^) was significantly related to branch diameter in ash (*y* = 30.1.62–22.34 *x*, se = 7.65, *r*^2^ = 0.31, *P* = 0.010) but not in oak (Figure 6B).

**Figure 7:**
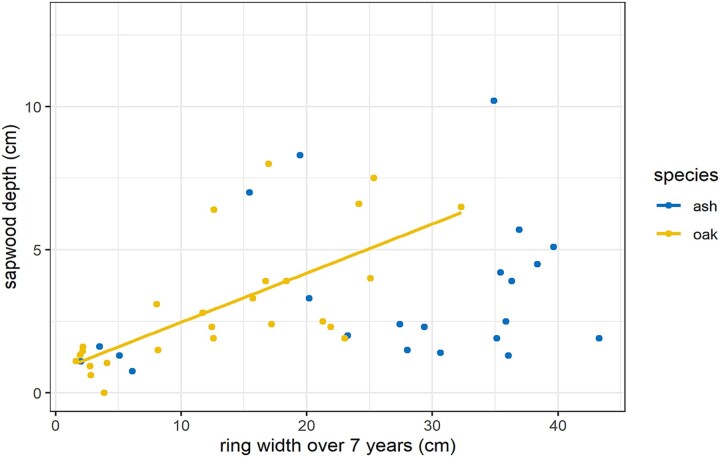
Sapwood depth as a function of the average of seven ring widths from one core, data here pertain to the vertical sample. The OLS regression slope is significant for oak but not for ash.

In oak but not ash, ring width was significantly related to sapwood depth by OLS regression (*y* = 4.78 + 2.799 *x*, se = 0.594, *r*^2^ = 0.45, *P* = < 0.001) with branches with greater ring width having deeper sapwood on average (Figure 7).

## Discussion

### Diameter-dependent variation of respiration

Woody-tissue respiration rates vary substantially within individuals and across branch diameters, consistent with prior findings (Damesin et al. 2002, Cavaleri et al. 2006, McGuire et al. 2007). Both surface area and volume significantly predict respiration, but yield contrasting flux patterns: Rt^area^ peaks in medium-to-large branches (Figure 2B), whereas Rt^vol^ is highest in small-diameter branches and declines asymptotically with increasing diameter (Figure 2A), in line with patterns observed in *Fagus sylvatica* (Damesin et al. 2002).

Although both metrics are informative, Rt^vol^ provides more robust empirical models, explaining greater variation in conjunction with branch diameter than Rt^area^ (Figures 2A and 4B). This suggests that respiration is largely surface-associated, with high Rt^vol^ values where surface-to-volume ratios are greatest. However, in branches <5 cm, elevated variability in Rt^vol^ implies a diminished association with surface area, indicating a greater role for volume-based flux. The rise in Rt^area^ up to ~10–20 cm diameter suggests initial volume-scaling of respiration, plateauing as sapwood volume ceases to increase proportionally with total volume and thus marking a shift to surface area scaling. This variation in scaling reflects underlying anatomical changes: larger branches contain substantial non-respiring heartwood, while respiring tissues (sapwood, cambium) form a thin surface layer. In smaller branches, where sapwood dominates, maintenance respiration aligns more closely with volume. Nonetheless, area-scaling persists to some extent due to the cambium’s proximity to the surface, as growth respiration remains localized to this thin, active tissue layer.

### Respiration, sapwood content and ring-width

Sapwood content is a known determinant of maintenance respiration. In our study, sapwood depth showed predictive power for CO₂ efflux, particularly when the growth component was excluded, improving model performance in ash but not in oak (Figures 3D and 6A). However, scaling respiration by sapwood volume (Rt^sap-vol^) is challenging due to measurement difficulty and its variation with branch diameter (Figure 3C). We also observe distinct species-specific sapwood development: ash exhibits substantially deeper sapwood in large branches than oak, reflecting divergent ecological strategies. Greater sapwood content in ash likely enhances water transport and supports higher photosynthetic and growth rates during the growing season. This relationship may explain the positive association between sapwood depth and ring width in oak (Figure 7), underscoring the importance of ecological strategy and associated physiology in shaping flux scaling.

While growth metabolism is known to drive temporal and inter-individual variation in respiration, few studies have examined the impact of intra-individual growth variation on CO₂ efflux. We find that Rt^area^ correlates strongly with ring width both within and between individuals of ash and oak (Figure 3B). Ring width was lowest in small branches and highest around 20 cm diameter, with increases more pronounced in ash, producing a greater difference in average ring width between small and medium branches compared with oak (Figure 3A). This pattern of growth and its species-specific expression is highly relevant for future studies of carbon accumulation in temperate forests. Our findings align with Han et al. (2017) and Tarvainen et al. (2014), who observed vertical gradients in temperature-compensated respiration in *Styphnolobium japonicum* and *Picea abies*, explained by greater diameter increments at higher stem positions. Han et al. (2017) reported daily diameter increments at 3.7 m that were twice those at 1.3 m, comparable to our own observations over similar height ranges.

### Diameter-dependent variation of growth and maintenance respiration

Estimates of mean growth and maintenance respiration derived from regression and the metabolic cost of growth were broadly consistent across methods and species (Table 1), and agree with previous studies (Damesin et al. 2002, Meir and Grace 2002, Rodríguez-Calcerrada et al. 2019). However, both methods diverged with regard to the width of their confidence intervals. Whilst the construction-cost method yielded only moderate, biologically plausible uncertainty, the regression method produced highly uncertain estimates, with confidence intervals extending below zero or above 100%, reflecting the uncertainty in the regression slope resulting from variation in growth rates and CO_2_ efflux in our sample. Similarly, these wide confidence intervals reflect the fact that these annual and seasonal estimates incorporate uncertainty from the temperature normalization (*Q*₁₀), dendrometer-based growth scaling, and model residual variance and should be interpreted as illustrative up-scales for these stands in 2017.

Both approaches show a larger contribution of growth respiration in ash, consistent with its higher growth rates during the study period (June–July). Maintenance respiration per unit sapwood was also higher in ash (Figures 4A and 5A), indicating a generally higher metabolic rate (Spicer and Holbrook 2007). Scaling relationships of component fluxes (expressed per area and per volume) followed similar trends with branch diameter as total flux. Therefore, by the logic outlined in paragraph 3 of the ‘Data analysis’ subsection of the methods, both growth and maintenance respiration appear primarily associated with surface area, except in branches <5 cm diameter, where volume becomes more relevant. These results inform seasonal scaling of woody flux and have implications for ecosystem-level estimates. Notably, relative rates of growth respiration per unit area increased with declining diameter, peaking at ~170% of DBH in oak and ~200% in ash at ~20 cm diameter (Figure S5 available as Supplementary Data at *Tree Physiology* Online, for GAM outputs see Table S8 available as Supplementary Data at *Tree Physiology* Online), before declining sharply to ~1–5% of DBH in the smallest branches. These findings emphasize that relying solely on measurements at breast height (1.3 m) may misrepresent the contributions of growth and maintenance respiration, leading to erroneous tree and ecosystem-scale estimates.

A difference was apparent in the methods used to estimate diameter increment, with ring widths yielding smaller growth estimates than dendrometers (Figure S3 available as Supplementary Data at *Tree Physiology* Online). This discrepancy likely reflects both physiological and methodological factors, as ring widths capture only permanent xylem accumulation, whereas dendrometer measurements also include reversible, hydration-driven stem dynamics and are subject to additional measurement biases associated with converting imperfect circumferential changes to diameter increments. These factors also explain the relatively low growth at DBH inferable from Figure 4 as compared with Table 1, as the former are based on wood cores. In addition, Figure 4 includes only the subset of climbed trees, which were selected for size and accessibility, potentially biasing the sample towards larger, slower-growing individuals.

Our finding that growth, maintenance and total respiration fluxes scale similarly with diameter (Figures 4 and 5) contrasts with previous studies reporting differential scaling between growth and maintenance respiration across seasons (Darenova et al. 2020). These discrepancies may reflect physiological differences, such as deeper sapwood or a broader distribution of active parenchyma, which could limit surface area scaling to the growth component alone. However, several methodological limitations must also be acknowledged. Although accurately partitioning growth and maintenance respiration remains an unresolved challenge (Salomón et al. 2024) and our assumptions are consistent with those used in earlier studies (Meir and Grace 2002, Salomón et al. 2024), a key limitation is that while we directly measured tree-level growth, seasonal scaling was derived from species-level growth rates rather than individual trajectories. Continuous monitoring using dendrometer bands would improve resolution and accuracy. Furthermore, both methods for estimating growth respiration have limitations; the construction cost method relies on a theoretical coefficient for the metabolic demand of wood synthesis, which may vary among species with different ecologies and may not capture metabolic costs associated with tissue support, such as elevated phloem activity (Rodríguez-Calcerrada et al. 2019).

The absence of a winter field campaign also limits our ability to assess whether scaling with diameter and temperature differs in the dormant season, as reported by Salomón et al. (2022). Although the lack of nighttime measurements may bias growth estimates, as stem growth has been reported to occur predominantly at night (Zweifel et al. 2021), the absence of consistent diel patterns in stem CO₂ efflux (Katayama et al. 2016, Mills et al. 2025), documented daytime growth in well-watered forests (Mencuccini et al. 2017) and the strong associations observed here between growth metrics and daytime efflux suggest that daytime measurements retain meaningful biological signal, particularly for analyses of within-tree variation. Furthermore, given that species average growth rates are generally used in ecosystem estimates of this flux, the models and data presented here provide a significant advance, allowing for the modelling of woody-tissue respiration with reduced uncertainty related to structural variation.

Although variation in growth and maintenance respiration accounts for much of the observed pattern in woody CO₂ efflux, additional factors likely contribute. As shown in Figure 6B, Rtm^sap-vol^ remains significantly and negatively related to branch diameter in ash, with smaller branches exhibiting higher average efflux despite similar sapwood content. One possible explanation is the contribution of translocated CO₂ originating from soil, roots and lower stem tissues, which dissolves in xylem sap and moves upward in the transpiration stream before diffusing radially through the bark (Bowman et al. 2005, Salomón et al. 2016, 2024). Prior research has found greater bark CO₂ efflux from smaller, upper branches attributed to shorter diffusive pathways from sapwood to atmosphere (Hölttä and Kolari 2009, Salomón et al. 2021). Another plausible explanation is that sapwood depth alone may not determine maintenance respiration as strongly as the parenchyma content within it. This fraction is highly variable and may differ systematically between trunks and branches of varying sizes (Carlquist 2001, Ziemińska et al. 2013, Rodríguez-Calcerrada et al. 2015), potentially leading to differing efflux rates even where sapwood depth is similar.

Increased variation in Rt^vol^ at the smallest diameters may be driven by several extraneous factors. Growth rates likely contribute, as distal branches are influenced by the local light environment—enhanced by light foraging and suppressed by shading. Similarly, spatial variability of canopy lighting might influence rates of corticular photosynthesis, of which the strongest evidence of appreciable metabolic contributions is derived from studies of twigs and juvenile stems (Pfanz et al. 2002). Therefore, in these smallest branch samples, there is likely a variable and unquantified proportion of photosynthetically active cells with potential to affect measured CO_2_ efflux directly, by their potentially higher specific respiration rates, and indirectly by the refixation of sap-dissolved CO_2_ (Ávila et al. 2014). Finally, nutrient concentrations in wood can vary within a tree, often being higher in branches and upper stems than near the base (Inagawa et al. 2023). This spatial heterogeneity provides a potential mechanism by which substrate availability may influence respiration rates of wood in the canopy.

Variation among individuals at DBH revealed that CO₂ efflux was inversely related to stem diameter in both species, though the signal was noisier at this scale as compared with the variation observed across the stems and crowns within trees. In ash, this relationship was evident using Rt^vol^, whereas in oak it only became significant after accounting for growth and isolating maintenance respiration. This supports the notion that efflux is more strongly associated with surface area than volume, with smaller diameters exhibiting higher volume-normalized fluxes due to a greater surface-to-volume ratio. Although scaling patterns were broadly similar within and between individuals, the between-tree dataset was markedly noisier, suggesting stronger extraneous CO₂ sources among trees. For example, translocated CO₂, influenced by microclimate and competitive interactions, may affect stem fluxes more between than within individuals (Bowman et al. 2005). These background effects likely obscure the respiration signal in oak more than in ash, due to oak’s lower flux rates and narrower DBH range. Species differences were evident in the sensitivity of maintenance respiration to DBH, reflected in differing slopes and intercepts for ash and oak. While rates were similar at larger DBH, ash exhibited higher efflux at smaller DBH. This may reflect species-specific differences in sapwood depth or growth rates. Given that ash grows faster than oak, individuals of the same DBH are likely younger and may exhibit higher metabolic rates, as older cells tend to respire less (Spicer, 2014).

## Conclusions

In conclusion, our findings provide a detailed view of how woody-tissue respiration varies by diameter within trees and among individuals, emphasizing the complex interplay between structural characteristics, tissue composition and species-specific growth strategies. Respiration rates were primarily associated with surface area, particularly in larger branches, but volume scaling became increasingly important in smaller diameters where sapwood dominated. Differences between oak and ash highlight the influence of ecological strategy and associated physiology on respiration dynamics, with ash displaying higher growth and maintenance respiration linked to deeper sapwood and faster growth. Although scaling relationships were generally consistent across growth and maintenance components, significant noise, especially in small branches and between individuals, underscores the importance of considering additional factors such as translocated CO₂, parenchymal variability and local environmental influences. These insights are critical for refining models of carbon cycling at tree and ecosystem scales, and future studies should aim to reduce methodological uncertainties, particularly around individual growth monitoring and sapwood characterization, to further improve the accuracy of scaling the woody flux.

## Supplementary Material

walker_2026_supplement_wytham_respiration_tpag060

## Data Availability

Please contact the corresponding author for data and materials.
