# Supplementary material for: Diameter-driven variation in wood CO2 efflux across the stems and crowns of three temperate broadleaf tree species
Source: Tree Physiol. 2026 May 7;46(6):tpag060. doi: 10.1093/treephys/tpag060 (PMC13264429; doi:10.1093/treephys/tpag060)
Supplement: walker_2026_supplement_wytham_respiration_tpag060 [file walker_2026_supplement_wytham_respiration_tpag060.docx]

Diameter-driven variation in wood CO₂ efflux across the stems and crowns of three temperate broadleaf tree species: Supplementary material

Tables:

| **Table S1**: raw data for intensively sampled trees including height and date/time data. | | | | | | | | | | |
| --- | --- | --- | --- | --- | --- | --- | --- | --- | --- | --- |
| tree number | height  (m) | order | CO_2_  efflux  µmol m^-2^ s^-1^  (uncorrected) | DBH  (cm) | Diameter  (cm) | stem temperature  (°C) | air temperature  (°C) | species | date | time |
| 4 | 24.5 | 7 | 1.77 | 78.62 | 2.9 | 25.7 | 24.5 | syc | 26/06/2017 | 10:46 |
| 4 | 21.5 | 6 | 2.08 | 78.62 | 18.5 | 22.8 | 21.3 | syc | 26/06/2017 | 11:22 |
| 4 | 19.1 | 5 | 2.02 | 78.62 | 27.7 | 22.5 | 21.3 | syc | 26/06/2017 | 11:56 |
| 4 | 16.5 | 4 | 2.65 | 78.62 | 33.4 | 21.9 | 20.2 | syc | 26/06/2017 | 12:27 |
| 4 | 12.2 | 3 | 2.02 | 78.62 | 49.3 | 21.5 | 19.9 | syc | 26/06/2017 | 13:09 |
| 4 | 9.1 | 2 | 1.70 | 78.62 | 54.1 | 23 | 18.9 | syc | 26/06/2017 | 13:53 |
| 4 | 6.6 | 1 | 2.08 | 78.62 | 65.6 | 22.3 | 18.9 | syc | 26/06/2017 | 14:21 |
| 4 | 1.3 | 0 | 2.08 | 78.62 | 78.6 | 23.7 | 21.4 | syc | 26/06/2017 | 14:44 |
| 6 | 21 | 8 | 0.95 | 142.6 | 1.5 | 25 | 24.7 | syc | 28/06/2017 | 10:31 |
| 6 | 19.3 | 7 | 2.08 | 142.6 | 13.7 | 21 | 19.3 | syc | 28/06/2017 | 11:12 |
| 6 | 19.1 | 6 | 2.08 | 142.6 | 17.8 | 20.3 | 19.9 | syc | 28/06/2017 | 11:51 |
| 6 | 18.2 | 5 | 3.16 | 142.6 | 22 | 21.5 | 20.4 | syc | 28/06/2017 | 12:27 |
| 6 | 16.6 | 4 | 2.84 | 142.6 | 24.8 | 20.3 | 20 | syc | 28/06/2017 | 12:59 |
| 6 | 15.5 | 3 | 2.08 | 142.6 | 36.3 | 20.7 | 20.4 | syc | 28/06/2017 | 13:41 |
| 6 | 12.5 | 2 | 1.70 | 142.6 | 43.3 | 20.7 | 20.7 | syc | 28/06/2017 | 14:25 |
| 6 | 10.1 | 1 | 1.89 | 142.6 | 50.6 | 21.9 | 21.9 | syc | 28/06/2017 | 15:03 |
| 6 | 1.3 | 0 | 1.89 | 142.6 | 68.8 | 21.6 | 21.6 | syc | 28/06/2017 | 15:35 |
| 2 | 23 | 8 | 0.38 | 101.54 | 2.3 | 26.1 | 26.6 | ash | 21/06/2017 | 10:40 |
| 2 | 19.9 | 7 | 8.84 | 101.54 | 29 | 25.7 | 25.1 | ash | 21/06/2017 | 11:19 |
| 2 | 18.1 | 6 | 5.05 | 101.54 | 36.3 | 26.9 | 25 | ash | 21/06/2017 | 11:57 |
| 2 | 16.1 | 5 | 6.38 | 101.54 | 49 | 27.3 | 24 | ash | 21/06/2017 | 12:24 |
| 2 | 14.5 | 4 | 6.31 | 101.54 | 54.4 | 29.2 | 24 | ash | 21/06/2017 | 13:08 |
| 2 | 12.8 | 3 | 6.19 | 101.54 | 62.4 | 26.1 | 24.8 | ash | 21/06/2017 | 13:53 |
| 2 | 9.9 | 2 | 6.76 | 101.54 | 76.4 | 25.8 | 25.7 | ash | 21/06/2017 | 14:33 |
| 2 | 7.5 | 1 | 5.24 | 101.54 | 124.1 | 26.1 | 24.4 | ash | 21/06/2017 | 15:01 |
| 2 | 1.3 | 0 | 5.68 | 101.54 | 101.5 | 24.9 | 23.6 | ash | 21/06/2017 | 15:42 |
| 8 | 21 | 7 | 0.25 | 49.33 | 1.9 | 22 | 19.6 | ash | 30/06/2017 | 11:04 |
| 8 | 18.9 | 6 | 5.24 | 49.33 | 15 | 21.8 | 18.9 | ash | 30/06/2017 | 11:39 |
| 8 | 17.7 | 5 | 7.32 | 49.33 | 19.4 | 20.6 | 19 | ash | 30/06/2017 | 12:17 |
| 8 | 16.3 | 4 | 8.33 | 49.33 | 21.3 | 22.5 | 20.2 | ash | 30/06/2017 | 12:42 |
| 8 | 14.9 | 3 | 0.00 | 49.33 | 23.6 | 21.1 | 20.5 | ash | 30/06/2017 | 13:16 |
| 8 | 12.7 | 2 | 4.73 | 49.33 | 26.7 | 21.3 | 20.8 | ash | 30/06/2017 | 13:48 |
| 8 | 9.2 | 1 | 7.07 | 49.33 | 42.7 | 21.6 | 20.9 | ash | 30/06/2017 | 14:24 |
| 8 | 1.3 | 0 | 8.84 | 49.33 | 49.3 | 21.8 | 21.5 | ash | 30/06/2017 | 15:05 |
| 9 | 21 | 8 | 0.51 | 57.29 | 0.9 | 23 | 22.5 | ash | 02/07/2017 | 10:44 |
| 9 | 17.6 | 7 | 4.10 | 57.29 | 11.1 | 24.9 | 24 | ash | 02/07/2017 | 11:26 |
| 9 | 16.4 | 6 | 6.19 | 57.29 | 14 | 21.9 | 23.4 | ash | 02/07/2017 | 12:06 |
| 9 | 15.4 | 5 | 8.33 | 57.29 | 18.1 | 23.7 | 26.4 | ash | 02/07/2017 | 12:37 |
| 9 | 13.7 | 4 | 7.20 | 57.29 | 22.9 | 24.8 | 22.2 | ash | 02/07/2017 | 13:13 |
| 9 | 12.6 | 3 | 7.58 | 57.29 | 29.6 | 21 | 22.2 | ash | 02/07/2017 | 13:55 |
| 9 | 8.5 | 2 | 7.77 | 57.29 | 34.1 | 23 | 22.4 | ash | 02/07/2017 | 14:41 |
| 9 | 6.9 | 1 | 8.71 | 57.29 | 44.9 | 23.7 | 21.5 | ash | 02/07/2017 | 15:10 |
| 9 | 1.3 | 0 | 4.92 | 57.29 | 57.3 | 23.6 | 20.8 | ash | 02/07/2017 | 15:44 |
| 5 | 19 | 7 | 0.44 | 70 | 1.9 | 21.4 | 19.6 | oak | 27/06/2017 | 10:55 |
| 5 | 16.4 | 6 | 1.07 | 70 | 13.1 | 20.4 | 18.1 | oak | 27/06/2017 | 11:39 |
| 5 | 15.4 | 5 | 3.16 | 70 | 17.5 | 21.5 | 18.6 | oak | 27/06/2017 | 12:14 |
| 5 | 13.7 | 4 | 2.15 | 70 | 21.3 | 19.1 | 17.8 | oak | 27/06/2017 | 12:51 |
| 5 | 12.6 | 3 | 2.71 | 70 | 28.3 | 20.6 | 18.8 | oak | 27/06/2017 | 13:35 |
| 5 | 8.5 | 2 | 2.65 | 70 | 34.1 | 20.9 | 19.6 | oak | 27/06/2017 | 14:01 |
| 5 | 6.9 | 1 | 3.28 | 70 | 68.1 | 20 | 18 | oak | 27/06/2017 | 14:37 |
| 5 | 1.3 | 0 | 2.53 | 70 | 70 | 20 | 18 | oak | 27/06/2017 | 15:25 |
| 3 | 19 | 6 | 1.64 |  | 1 | 23.2 | 21.6 | oak | 23/06/2017 | 11:13 |
| 3 | 16 | 5 | 2.65 | 76.39 | 16.2 | 23.2 | 21.8 | oak | 23/06/2017 | 11:52 |
| 3 | 15 | 4 | 3.79 | 76.39 | 24.2 | 23.6 | 23.3 | oak | 23/06/2017 | 12:35 |
| 3 | 13.5 | 3 | 3.22 | 76.39 | 33.4 | 23.2 | 23.3 | oak | 23/06/2017 | 13:11 |
| 3 | 10.6 | 2 | 3.03 | 76.39 | 54.1 | 22.2 | 22.2 | oak | 23/06/2017 | 13:49 |
| 3 | 8.4 | 1 | 1.33 | 76.39 | 66.2 | 21.5 | 20.7 | oak | 23/06/2017 | 14:32 |
| 3 | 1.3 | 0 | 1.14 | 76.39 | 76.4 | 20.6 | 20.3 | oak | 23/06/2017 | 15:15 |
| 7 | 15 | 8 | 0.19 | 64.29 | 1.8 | 23.2 | 21.2 | oak | 29/06/2017 | 10:51 |
| 7 | 12.8 | 7 | 4.17 | 64.29 | 13.7 | 19.3 | 18.3 | oak | 29/06/2017 | 11:33 |
| 7 | 11.6 | 6 | 4.17 | 64.29 | 14.6 | 20.4 | 18.9 | oak | 29/06/2017 | 12:15 |
| 7 | 10.7 | 5 | 3.09 | 64.29 | 19.7 | 21.5 | 20.3 | oak | 29/06/2017 | 13:07 |
| 7 | 10 | 4 | 3.41 | 64.29 | 23.6 | 21.3 | 20.5 | oak | 29/06/2017 | 13:46 |
| 7 | 7.5 | 3 | 4.17 | 64.29 | 33.7 | 21.4 | 20.5 | oak | 29/06/2017 | 14:19 |
| 7 | 6 | 2 | 1.33 | 64.29 | 49.7 | 20.6 | 20.4 | oak | 29/06/2017 | 14:59 |
| 7 | 3.6 | 1 | 1.89 | 64.29 | 65.3 | 20.1 | 19.4 | oak | 29/06/2017 | 15:41 |
| 7 | 1.3 | 0 | 1.70 | 64.29 | 64.3 | 21.5 | 21.4 | oak | 29/06/2017 | 16:13 |
| 1 | 23 | 8 | 0.51 | 127.32 | 0.5 | 24.9 | 25.4 | syc | 19/06/2017 | 13:14 |
| 1 | 21.5 | 7 | 1.70 | 127.32 | 13.2 | 22.9 | 21.9 | syc | 20/06/2017 | 13:13 |
| 1 | 18.8 | 6 | 3.85 | 127.32 | 31.2 | 20.9 | 21 | syc | 20/06/2017 | 12:25 |
| 1 | 17.8 | 5 | 2.78 | 127.32 | 9.8 | 20.8 | 20.6 | syc | 20/06/2017 | 11:44 |
| 1 | 15.4 | 4 | 2.53 | 127.32 | 22.3 | 21.8 | 20.6 | syc | 20/06/2017 | 11:05 |
| 1 | 13.5 | 3 | 3.47 | 127.32 | 21.3 | 22.2 | 19.2 | syc | 19/06/2017 | 16:40 |
| 1 | 9.8 | 2 | 4.04 | 127.32 | 59.5 | 23 | 21.1 | syc | 19/06/2017 | 15:52 |
| 1 | 7.6 | 1 | 2.27 | 127.32 | 69.4 | 22.2 | 21.8 | syc | 19/06/2017 | 15:17 |
| 1 | 1.3 | 0 | 1.14 | 127.32 | 127.3 | 23 | 22.8 | syc | 19/06/2017 | 14:31 |

| **Table S2 A - D:** GAM modelling outputs of Rt^vol^ as a function of branch diameter for oak, ash, sycamore and all species combined. | | | | | | | | | | | | |  |  |
| --- | --- | --- | --- | --- | --- | --- | --- | --- | --- | --- | --- | --- | --- | --- |
| **A - GAM of ash Rt^vol^ as function of diameter** | | | | | | | | | | | | |  |  |
| Component | Term | | Estimate | | Std Error | | t-value | | p-value | |  | |  |  |
| A. parametric coefficients | (Intercept) | | 163.674 | | 10.989 | | 14.894 | | 0.0000 | | *** | |  |  |
| Component | Term | | edf | | Ref. df | | F-value | | p-value | |  | |  |  |
| B. smooth terms | s(diameter) | | 2.336 | | 2.878 | | 28.625 | | 0.0000 | | *** | |  |  |
| Adjusted R-squared: 0.759, Deviance explained 0.781, GCV : 3720.468, Scale est: 3260.758, N: 27 | | | | | | | | | | | | |  |  |
| **B - GAM of oak Rt^vol^ as function of diameter** | | | | | | | | | | | | |  |  |
| Component | Term | | Estimate | | Std Error | | t-value | | p-value | |  | |  |  |
| A. parametric coefficients | (Intercept) | | 106.494 | | 11.090 | | 9.603 | | 0.0000 | | *** | |  |  |
| Component | Term | | edf | | Ref. df | | F-value | | p-value | |  | |  |  |
| B. smooth terms | s(diameter) | | 1.860 | | 2.219 | | 17.498 | | 0.0000 | | *** | |  |  |
| Adjusted R-squared: 0.567, Deviance explained 0.595, GCV : 4078.259, Scale est: 3689.462, N: 30 | | | | | | | | | | | | |  |  |
| **C - GAM of sycamore Rt^vol^ as function of diameter** | | | | | | | | | | | | |  |  |
| Component | Term | | Estimate | | Std Error | | t-value | | p-value | |  | |  |  |
| A. parametric coefficients | (Intercept) | | 102.794 | | 9.771 | | 10.521 | | 0.0000 | | *** | |  |  |
| Component | Term | | edf | | Ref. df | | F-value | | p-value | |  | |  |  |
| B. smooth terms | s(diameter) | | 2.878 | | 3.493 | | 17.021 | | 0.0000 | | *** | |  |  |
| Adjusted R-squared: 0.626, Deviance explained 0.657, GCV : 3851.693, Scale est: 3436.773, N: 36 | | | | | | | | | | | | |  |  |
| **D - GAM of oak, ash and sycamore Rt^vol^ as function of diameter** | | | | | | | | | | | | | | |
| Component | | | Term | | Estimate | | Std Error | | t-value | | p-value | |  | |
| A. parametric coefficients | | | (Intercept) | | 175.951 | | 11.523 | | 15.270 | | 0.0000 | | *** | |
|  |  |  | speciesoak | | -73.495 | | 15.932 | | -4.613 | | 0.0000 | | *** | |
|  |  |  | speciessyc | | -79.001 | | 15.294 | | -5.166 | | 0.0000 | | *** | |
| Adjusted R-squared: 0.671, Deviance explained 0.690, GCV : 3787.800, Scale est: 3535.466, N: 93 | | | | | | | | | | | | | |  |
|  | | | | | | | | | | | | | |  |

| **Table S3 A - D:** GAM modelling outputs of Rt^area^ as a function of branch diameter for oak, ash, sycamore and all species combined. | | | | | | |
| --- | --- | --- | --- | --- | --- | --- |
| **A - GAM of ash Rt^area^ as function of diameter** | | | | | | |
| Component | Term | Estimate | Std Error | t-value | p-value |  |
| A. parametric coefficients | (Intercept) | 4.671 | 0.268 | 17.400 | 0.0000 | *** |
| Component | Term | edf | Ref. df | F-value | p-value |  |
| B. smooth terms | s(diameter) | 3.732 | 3.957 | 14.053 | 0.0000 | *** |
| Adjusted R-squared: 0.677, Deviance explained 0.722, GCV : 2.429, Scale est: 2.018, N: 28 | | | | | | |
| **B - GAM of oak Rt^area^ function of diameter** | | | | | | |
| Component | Term | Estimate | Std Error | t-value | p-value |  |
| A. parametric coefficients | (Intercept) | 2.199 | 0.164 | 13.411 | 0.0000 | *** |
| Component | Term | edf | Ref. df | F-value | p-value |  |
| B. smooth terms | s(diameter) | 2.718 | 2.935 | 11.535 | 0.0001 | *** |
| Adjusted R-squared: 0.483, Deviance explained 0.527, GCV : 1.000, Scale est: 0.887, N: 33 | | | | | | |
| **C - GAM of sycamore Rt^area^ as function of diameter** | | | | | | |
| Component | Term | Estimate | Std Error | t-value | p-value |  |
| A. parametric coefficients | (Intercept) | 1.798 | 0.128 | 13.999 | 0.0000 | *** |
| Component | Term | edf | Ref. df | F-value | p-value |  |
| B. smooth terms | s(diameter) | 2.790 | 2.964 | 9.749 | 0.0002 | *** |
| Adjusted R-squared: 0.419, Deviance explained 0.465, GCV : 0.664, Scale est: 0.594, N: 36 | | | | | | |
| **D - GAM of oak, ash and sycamore Rt^area^ as function of diameter** | | | | | | |
| Component | Term | Estimate | Std Error | t-value | p-value |  |
| A. parametric coefficients | (Intercept) | 4.483 | 0.227 | 19.773 | 0.0000 | *** |
|  | speciesoak | -2.220 | 0.311 | -7.143 | 0.0000 | *** |
|  | speciessyc | -2.596 | 0.303 | -8.578 | 0.0000 | *** |
| Component | Term | edf | Ref. df | F-value | p-value |  |
| B. smooth terms | s(diameter) | 5.189 | 6.395 | 14.501 | 0.0000 | *** |
| Adjusted R-squared: 0.671, Deviance explained 0.695, GCV : 1.519, Scale est: 1.391, N: 97 | | | | | | |
|  | | | | | | |

| **Table S4 A - C:** GAM modelling outputs of oak and ash ring width as a function of branch diameter for oak, ash and both species combined. | | | | | | |
| --- | --- | --- | --- | --- | --- | --- |
| **A - GAM of oak and ash rings as function of diameter** | | | | | | |
| Component | Term | Estimate | Std Error | t-value | p-value |  |
| A. parametric coefficients | (Intercept) | 0.225 | 0.010 | 22.171 | 0.0000 | *** |
|  | speciesoak | -0.117 | 0.014 | -8.372 | 0.0000 | *** |
| Component | Term | edf | Ref. df | F-value | p-value |  |
| B. smooth terms | s(diameter) | 2.963 | 2.999 | 36.714 | 0.0000 | *** |
| Adjusted R-squared: 0.832, Deviance explained 0.846, GCV : 0.00242, Scale est: 0.00217, N: 48 | | | | | | |
| **B - GAM of ash rings as function of diameter** | | | | | | |
| Component | Term | Estimate | Std Error | t-value | p-value |  |
| A. parametric coefficients | (Intercept) | 0.243 | 0.009 | 27.598 | 0.0000 | *** |
| Component | Term | edf | Ref. df | F-value | p-value |  |
| B. smooth terms | s(diameter) | 3.871 | 3.989 | 31.696 | -0.0000 | *** |
| Adjusted R-squared: 0.859, Deviance explained 0.885, GCV : 0.00219, Scale est: 0.0017, N: 22 | | | | | | |
| **C - GAM of oak rings as function of diameter** | | | | | | |
| Component | Term | Estimate | Std Error | t-value | p-value |  |
| A. parametric coefficients | (Intercept) | 0.092 | 0.005 | 16.964 | 0.0000 | *** |
| Component | Term | edf | Ref. df | F-value | p-value |  |
| B. smooth terms | s(diameter) | 2.901 | 2.992 | 29.100 | 0.0000 | *** |
| Adjusted R-squared: 0.767, Deviance explained 0.794, GCV : 0.000904, Scale est: 0.000768, N: 26 | | | | | | |
|  | | | | | | |

| **Table S5**: GAM modelling outputs of ash sapwood depth as a function of branch diameter. | | | | | | |
| --- | --- | --- | --- | --- | --- | --- |
| Component | Term | Estimate | Std Error | t-value | p-value |  |
| A. parametric coefficients | (Intercept) | 3.539 | 0.460 | 7.693 | 0.0000 | *** |
| Component | Term | edf | Ref. df | F-value | p-value |  |
| B. smooth terms | s(diameter) | 1.000 | 1.000 | 22.223 | 0.0001 | *** |
| Adjusted R-squared: 0.414, Deviance explained 0.434, GCV : 7.012, Scale est: 6.560, N: 31 | | | | | | |
|  | | | | | | |

| **Table S6**: GAM modelling outputs of oak and ash Rtm^vol^ and Rtg^vol^ as a function of branch diameter for oak, ash and both species combined. | | | | | | | |
| --- | --- | --- | --- | --- | --- | --- | --- |
| **A - GAM of oak Rtg^vol^ as function of diameter** | | | | | | | |
| Componentg | | Term | Estimate | Std Error | t-value | p-value |  |
| A. parametric coefficients | | (Intercept) | 79.753 | 10.161 | 7.849 | 0.0000 | *** |
| Component | | Term | edf | Ref. df | F-value | p-value |  |
| B. smooth terms | | s(diameter) | 1.000 | 1.000 | 21.565 | 0.0001 | *** |
| Adjusted R-squared: 0.472, Deviance explained 0.495, GCV : 2703.107, Scale est: 2477.848, N: 24 | | | | | | | |
| **B - GAM of oak Rtg^vol^ as function of diameter** | | | | | | |  |
| Component | Term | Estimate | Std Error | t-value | p-value |  |  |
| A. parametric coefficients | (Intercept) | 11.627 | 1.505 | 7.726 | 0.0000 | *** |  |
| Component | Term | edf | Ref. df | F-value | p-value |  |  |
| B. smooth terms | s(diameter) | 2.326 | 2.681 | 18.000 | 0.0000 | *** |  |
| Adjusted R-squared: 0.657, Deviance explained 0.690, GCV : 65.308, Scale est: 56.619, N: 25 | | | | | | |  |
| **C - GAM of ash Rtm^vol^ as function of diameter** | | | | | | |  |
| Component | Term | Estimate | Std Error | t-value | p-value |  |  |
| A. parametric coefficients | (Intercept) | 131.925 | 11.453 | 11.519 | 0.0000 | *** |  |
| Component | Term | edf | Ref. df | F-value | p-value |  |  |
| B. smooth terms | s(diameter) | 1.808 | 1.963 | 22.988 | 0.0000 | *** |  |
| Adjusted R-squared: 0.683, Deviance explained 0.713, GCV : 3051.970, Scale est: 2623.438, N: 20 | | | | | | |  |
| **D - GAM of ash Rtg^vol^ as function of diameter** | | | | | | |  |
| Component | Term | Estimate | Std Error | t-value | p-value |  |  |
| A. parametric coefficients | (Intercept) | 16.629 | 2.864 | 5.806 | 0.0000 | *** |  |
| Component | Term | edf | Ref. df | F-value | p-value |  |  |
| B. smooth terms | s(diameter) | 2.475 | 2.810 | 10.691 | 0.0005 | *** |  |
| Adjusted R-squared: 0.566, Deviance explained 0.617, GCV : 214.337, Scale est: 180.483, N: 22 | | | | | | |  |
| **E - GAM of oak and ash Rtg^vol^ as function of diameter** | | | | | | |  |
| Component | Term | Estimate | Std Error | t-value | p-value |  |  |
| A. parametric coefficients | (Intercept) | 17.748 | 2.444 | 7.263 | 0.0000 | *** |  |
|  | speciesoak | -7.104 | 3.358 | -2.116 | 0.0402 | * |  |
| Component | Term | edf | Ref. df | F-value | p-value |  |  |
| B. smooth terms | s(diameter) | 1.924 | 1.994 | 27.699 | 0.0000 | *** |  |
| Adjusted R-squared: 0.537, Deviance explained 0.566, GCV : 142.670, Scale est: 130.759, N: 47 | | | | | | |  |
| **F - GAM of oak and ash Rtm^vol^ as function of diameter** | | | | | | |  |
| Component | Term | Estimate | Std Error | t-value | p-value |  |  |
| A. parametric coefficients | (Intercept) | 135.039 | 11.733 | 11.509 | 0.0000 | *** |  |
|  | speciesoak | -57.880 | 15.919 | -3.636 | 0.0008 | *** |  |
| Component | Term | edf | Ref. df | F-value | p-value |  |  |
| B. smooth terms | s(diameter) | 1.721 | 2.117 | 26.248 | 0.0000 | *** |  |
| Adjusted R-squared: 0.600, Deviance explained 0.626, GCV : 2992.838, Scale est: 2739.766, N: 44 | | | | | | |  |
|  | | | | | | |  |

| **Table S7:** GAM modelling outputs of oak and ash Rtm^area^ and Rtg^area^ as a function of branch diameter for oak, ash and both species combined. | | | | | | |  |
| --- | --- | --- | --- | --- | --- | --- | --- |
| **A - GAM of oak Rtm^area^ as a function of diameter** | | | | | | |  |
| Component | Term | Estimate | Std Error | t-value | p-value |  |  |
| A. parametric coefficients | (Intercept) | 2.165 | 0.222 | 9.747 | 0.0000 | *** |  |
| Component | Term | edf | Ref. df | F-value | p-value |  |  |
| B. smooth terms | s(diameter) | 1.901 | 1.990 | 4.769 | 0.0169 | * |  |
| Adjusted R-squared: 0.272, Deviance explained 0.330, GCV : 1.396, Scale est: 1.234, N: 25 | | | | | | |  |
|  | | | | | | |  |
| **B - GAM of oak Rtg^area^ as function of diameter** | | | | | | |  |
| Component | Term | Estimate | Std Error | t-value | p-value |  |  |
| A. parametric coefficients | (Intercept) | 0.161 | 0.011 | 14.842 | 0.0000 | *** |  |
| Component | Term | edf | Ref. df | F-value | p-value |  |  |
| B. smooth terms | s(diameter) | 2.927 | 2.996 | 34.747 | 0.0000 | *** |  |
| Adjusted R-squared: 0.806, Deviance explained 0.830, GCV : 0.00349, Scale est: 0.00294, N: 25 | | | | | | |  |
| **C - GAM of ash Rtm^area^ as function of diameter** | | | | | | |  |
| Component | Term | Estimate | Std Error | t-value | p-value |  |  |
| A. parametric coefficients | (Intercept) | 4.297 | 0.405 | 10.612 | 0.0000 | *** |  |
| Component | Term | edf | Ref. df | F-value | p-value |  |  |
| B. smooth terms | s(diameter) | 1.818 | 1.967 | 3.815 | 0.0606 | . |  |
| Adjusted R-squared: 0.235, Deviance explained 0.308, GCV : 3.818, Scale est: 3.280, N: 20 | | | | | | |  |
|  | | | | | | |  |
| **D - GAM of ash Rtg^area^ as function of diameter** | | | | | | |  |
| Component | Term | Estimate | Std Error | t-value | p-value |  |  |
| A. parametric coefficients | (Intercept) | 0.350 | 0.018 | 18.969 | 0.0000 | *** |  |
| Component | Term | edf | Ref. df | F-value | p-value |  |  |
| B. smooth terms | s(diameter) | 2.966 | 2.999 | 29.056 | -0.0000 | *** |  |
| Adjusted R-squared: 0.800, Deviance explained 0.828, GCV : 0.00913, Scale est: 0.00749, N: 22 | | | | | | |  |
|  | | | | | | |  |
| **E- GAM of ash and oak Rtm^area^ as function of diameter** | | | | | | |  |
| Component | Term | Estimate | Std Error | t-value | p-value |  |  |
| A. parametric coefficients | (Intercept) | 4.239 | 0.333 | 12.713 | 0.0000 | *** |  |
|  | speciesoak | -2.027 | 0.448 | -4.525 | 0.0001 | *** |  |
| Component | Term | edf | Ref. df | F-value | p-value |  |  |
| B. smooth terms | | s(diameter) | 2.966 | 2.999 | 29.056 | -0.0000 | *** |
| Adjusted R-squared: 0.435, Deviance explained 0.472, GCV : 2.427, Scale est: 2.216, N: 45 | | | | | | |  |
|  | | | | | | | |
|  | | | | | | | |

| **Table S8:** GAM modelling outputs of Rtg^area^ relative to rates at DBH (ratio) as a function of branch diameter for oak, ash and both species combined. | | | | | | | |
| --- | --- | --- | --- | --- | --- | --- | --- |
| **A - GAM of percentage increase of oak Rtg^area^ relative to rates at DBH as function of diameter** | | | | | | | |
| Component | | Term | Estimate | Std Error | t-value | p-value |  |
| A. parametric coefficients | | (Intercept) | 140.913 | 12.106 | 11.640 | 0.0000 | *** |
| Component | | Term | edf | Ref. df | F-value | p-value |  |
| B. smooth terms | | s(diameter) | 2.790 | 2.966 | 8.920 | 0.0017 | ** |
| **B- GAM of percentage increase of ash Rtg^area^ relative to rates at DBH as function of diameter** | | | | | | |  |
| Component | Term | Estimate | Std Error | t-value | p-value |  |  |
| A. parametric coefficients | (Intercept) | 124.825 | 11.132 | 11.213 | 0.0000 | *** |  |
| Component | Term | edf | Ref. df | F-value | p-value |  |  |
| B. smooth terms | s(diameter) | 2.748 | 2.953 | 5.943 | 0.0106 | * |  |
| Adjusted R-squared: 0.418, Deviance explained 0.498, GCV : 3167.662, Scale est: 2602.262, N: 21 | | | | | | |  |
| **C - GAM of percentage increase of ash and oak Rtg^area^ relative to rates at DBH as function of diameter** | | | | | | |  |
| Component | Term | Estimate | Std Error | t-value | p-value |  |  |
| A. parametric coefficients | (Intercept) | 115.991 | 12.297 | 9.433 | 0.0000 | *** |  |
|  | speciesoak | 33.755 | 17.646 | 1.913 | 0.0635 | . |  |
| Component | Term | edf | Ref. df | F-value | p-value |  |  |
| B. smooth terms | s(diameter) | 2.920 | 2.995 | 10.811 | 0.0000 | *** |  |
| Adjusted R-squared: 0.436, Deviance explained 0.490, GCV : 3490.245, Scale est: 3081.419, N: 42 | | | | | | |  |
|  | | | | | | |  |

Figures


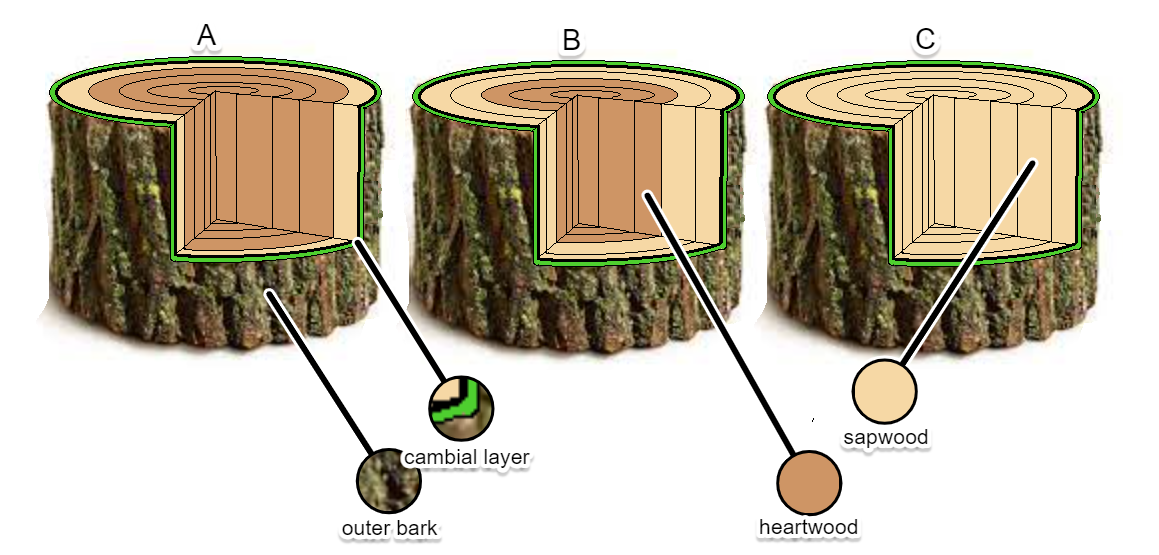


**Figure S1 (A):** a schematic of a branch or stem section more typical of older or larger samples, dominated by non-respiring heartwood, here sapwood forms a relatively thin layer above the heartwood and beneath the cambium. In samples like this area-based scaling is expected. **S1 (C) :** a schematic of a branch of stem section more typical of smaller or younger samples, here the sample is yet to develop heartwood and is dominated by sapwood. Volume based scaling is expected. **S1 (B):** a schematic of an intermediate branch or stem section in which the sapwood: heartwood ratio is roughly equal.


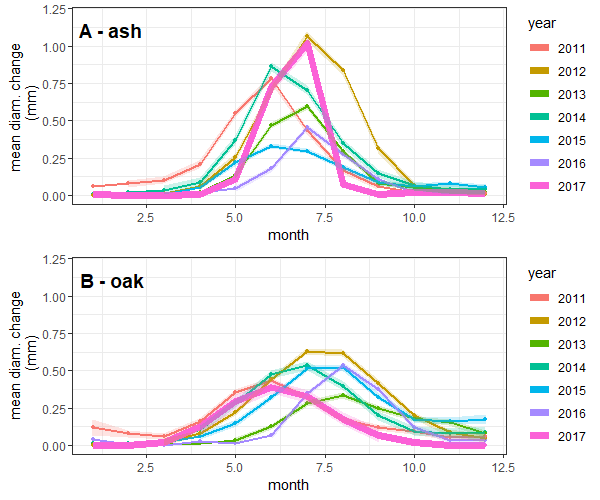


**Figure S2**: Seasonal growth rate of ash and oak as measured by dendrometer bands. Modelling monthly change of diameters at breast height (DBH) we see that ash trees on average have a more intense growing season, with greater increases observed at the peak of growing compared to oak. However, we also observe that the length of the growing season is longer in oak in comparison to ash. In both species we see some inter-annual variation in which month growth peaks.


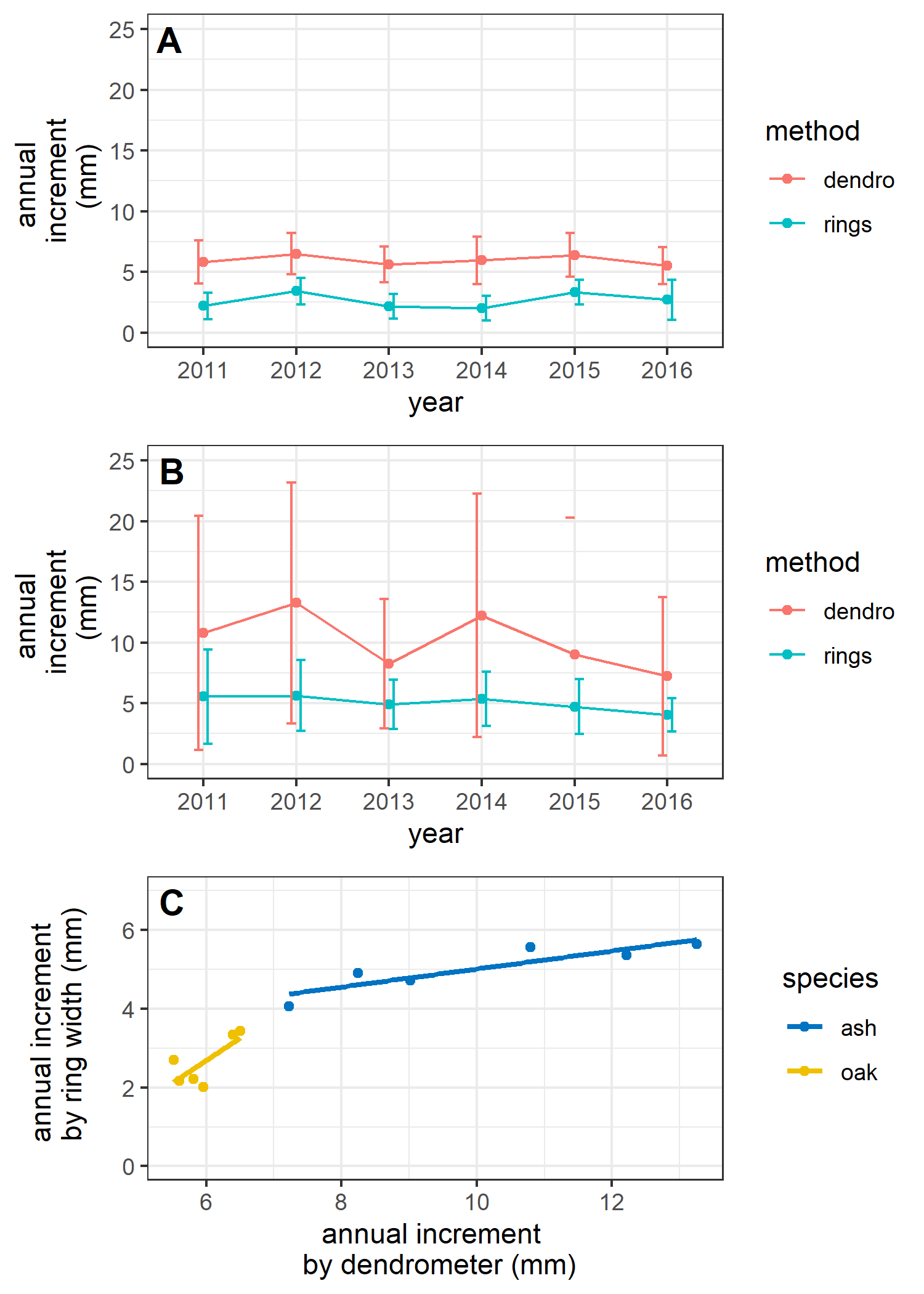


**Figure S3**: Interannual average total growth across the period of 2011 to 2016 as measured by dendrometers and by ring width for ash (**A**) and oak (**B**), each point here represents the total growth of a single tree in that year. (**C**) regression of dendrometer total growth estimates as a function of ring growth aligned by year.

It should be noted that 2017 is absent from Figure S3 because the ring-widths were determined from wood-cores collected early in the growing season, the outer ring corresponding to 2017 was very small, often damaged and therefore very difficult to measure. This is why we use the dendrometer data for the modelling work focussing on growth rates (and because it provides seasonality). The purpose of these graphs (S3 (A), (B) and (C)) is to demonstrate that in previous years dendrometer data can be used to predict ring-width data, indicating that in terms of total growth, our sample of respiration trees do not differ significantly from the sample for which we have dendrometer data.


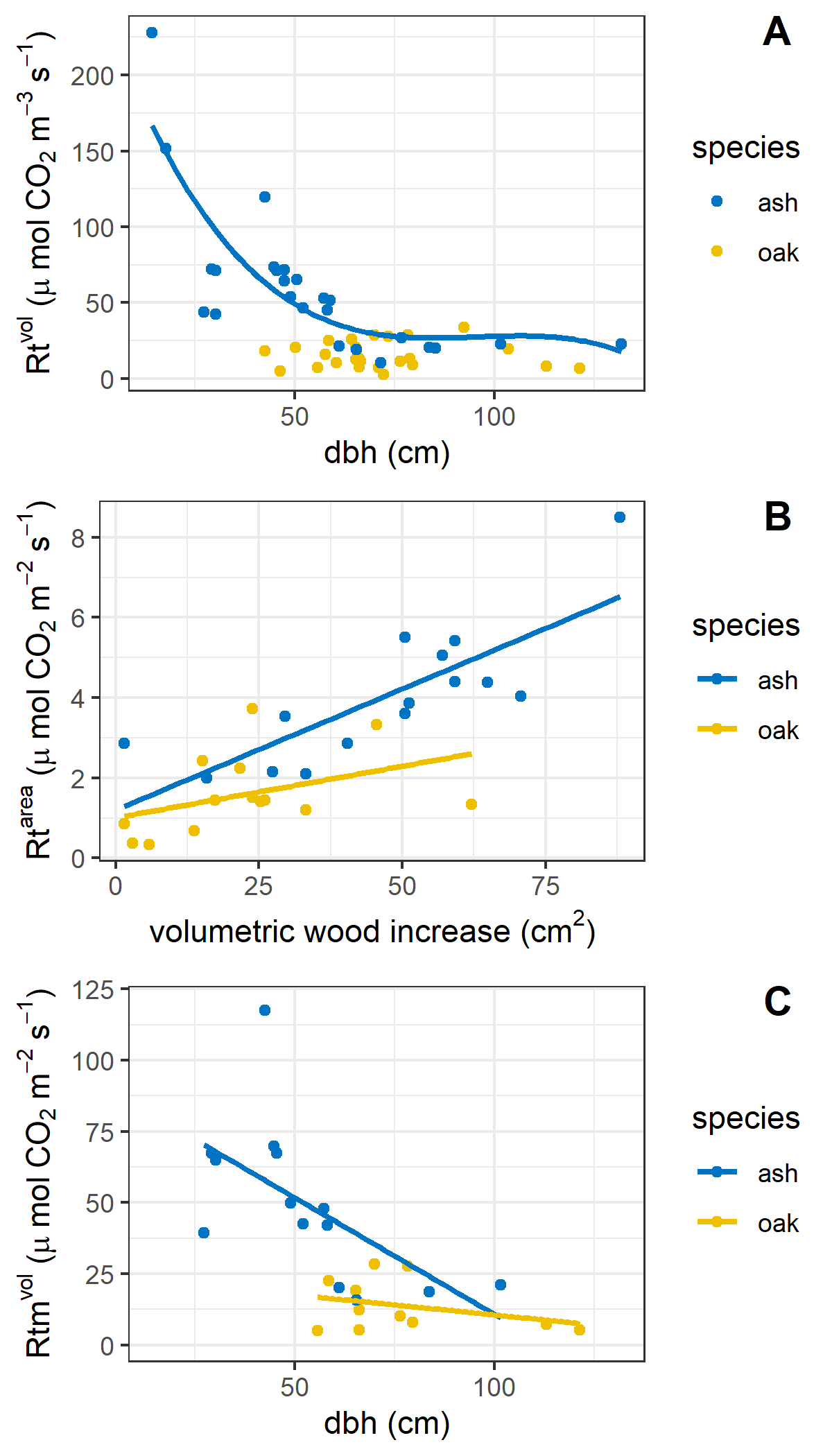


**Figure S4:** **A:** The relationship of Rt^vol^ as a function of DBH in oak and ash. Lines represent generalized additive models smooth function. **B:** Rt^area^ as a function of volumetric wood increase in ash and oak. **C:** Rtm^area^ as a function of DBH for ash and oak.

A significant negative effect of DBH on Rt^vol^, similar to that exhibited within the vertical transects, was found in the ash data set but not amongst oaks (Figure S5 A). However, using Rt^area^ signs of a weak signal, albeit non-significant, begin to appear in oak and using temperature-compensated maintenance respiration expressed volumetrically (Rmt^vol^ ) as calculated from the construction cost method, a significant effect of DBH was found for individual trees of both ash and oak and was improved by inclusion of species as an additive but not multiplicative predictor (y = 76.57 - 0.51, r^2^ = 0.56, p = <0.001) with the oak intercept being significantly lower than the model average (-22.86, p = 0.012).

From the sample of Ash and Oak individuals measured only at breast height, average rates of CO_2_ efflux were 2.91 +- 1.65 µmole m^-2^ s^-1^, ranging from 0.37- 7.19 µmole m^-2^ s^-1^ across diameters at breast height ranging from 14.32 to 131.78cm. As was the case within the vertical transect, ash trees at breast height had higher average rates of efflux than oak trees.

A significant effect of growth rate, as measured by the volumetric increase of wood over six years, was found on Rt^vol^ in ash and oak when tested by a multiple regression including species as an additive effect (y = 76.57 - 0.51, r^2^ = 0.56, p = <0.001) with the oak intercept being significantly lower than the model average (-22.86, p = 0.012). The intercepts of these linear models were taken as the sample average efflux at zero growth (i.e average maintenance respiration during the growing season).


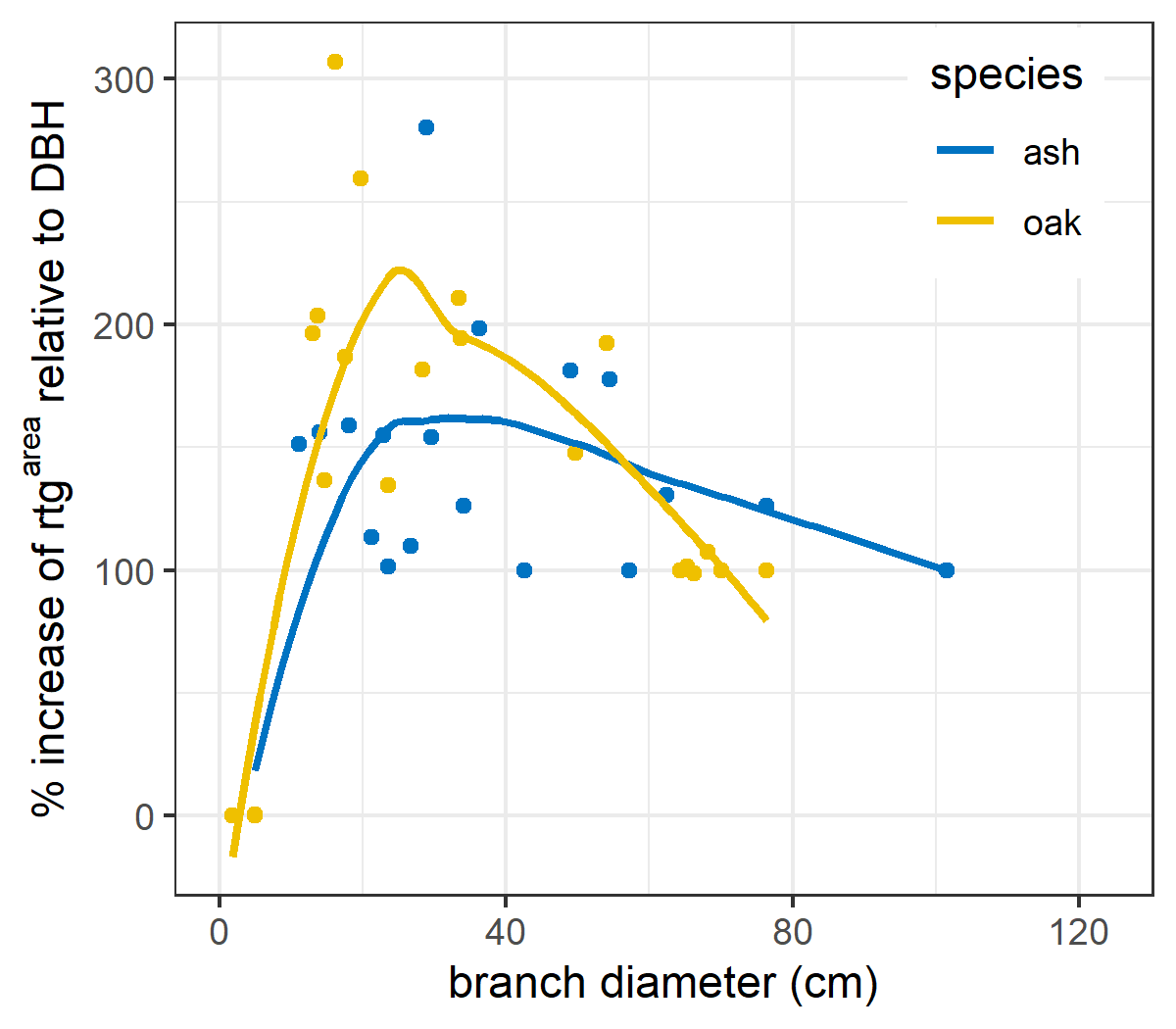


**Figure S5:** Percentage increase of Rtg^area^ relative to Rtg^area^ at DBH for intensively sampled trees. Expressing Rtg^area^ measurements as a percentage of the tree specific rate of efflux at DBH we can see that in branches ~10 – 30 cm diameter the growth component can be more than double the rate observed at DBH
